# Basal re-esterification finetunes mitochondrial fatty acid utilization

**DOI:** 10.1016/j.molmet.2023.101701

**Published:** 2023-03-04

**Authors:** Anand Kumar Sharma, Tongtong Wang, Alaa Othman, Radhika Khandelwal, Miroslav Balaz, Salvatore Modica, Nicola Zamboni, Christian Wolfrum

**Affiliations:** 1 Laboratory of Translational Nutrition Biology, Institute of Food, Nutrition and Health, ETH Zurich, Schwerzenbach, Switzerland; 2 Department of Biology, Institute of Molecular Systems Biology, ETH Zürich, Zürich, Switzerland; 3 Laboratory of Cellular and Molecular Metabolism, Biomedical Research Center, Slovak Academy of Sciences, Bratislava, Slovakia

**Keywords:** Adipose tissue, Lipolysis, Re-esterification, DGAT1, DGAT2, Fatty acid oxidation, Lipidomics, FA, Fatty acid, DGAT, Diacylglycerol O-acyltransferase, TAG, Triglyceride, LD, Lipid droplet, OCR, Oxygen consumption rate

## Abstract

**Objective:**

Emerging evidence suggest the existence of constant basal lipolysis and re-esterification of a substantial fraction of thus liberated fatty acids. In stimulated lipolysis, the re-esterification is proposed to be a protective mechanism against lipotoxicity; however, the role of the lipolysis coupled to re-esterification under basal conditions has not been deciphered.

**Methods:**

We used adipocytes (*in vitro* differentiated brown and white adipocytes derived from a cell line or primary SVF culture) to study the effect of inhibition of re-esterification by pharmacological DGAT1 and DGAT2 inhibitors alone or in combination. We then evaluated cellular energetics, lipolysis flux, and lipidomic parameters along with mitochondrial properties and fuel utilization.

**Results:**

In adipocytes, DGAT1 and 2 mediated re-esterification is a moderator of fatty acid oxidation. Combined inhibition of both DGATs (D1+2i) increases oxygen consumption, which is largely due to enhanced mitochondrial respiration by lipolysis-derived fatty acids (FAs). Acute D1+2i selectively affects mitochondrial respiration without affecting the transcriptional homeostasis of genes relevant to mitochondrial health and lipid metabolism. D1+2i enhances the mitochondrial import of pyruvate and activates AMP Kinase to counteract CPT1 antagonism, thus facilitating the mitochondrial import of fatty acyl-CoA.

**Conclusions:**

These data implicate the process of re-esterification in the regulation of mitochondrial FA usage and uncover a mechanism of FAO regulation via crosstalk with FA re-esterification.

## Highlights

• Inhibition of re-esterification increases basal OCR by diverting lipolysis-derived FAs to mitochondria.
• Unlike stimulated lipolysis when DGAT1 is dominant isofrom, at basal level two DGAT isozymes show compensatory activity.
• The adipocyte lipidome changes induced by DGATi are specific to some lipid species.
• AMPK activation by D1+2i causes a reduction in malonyl-CoA levels that facilitates increased mitochondrial import of FAs.

## Graphical abstract

Inhibition of basal re-esterification diverts FAs to mitochondria causing increase in OCR

**Figure:**
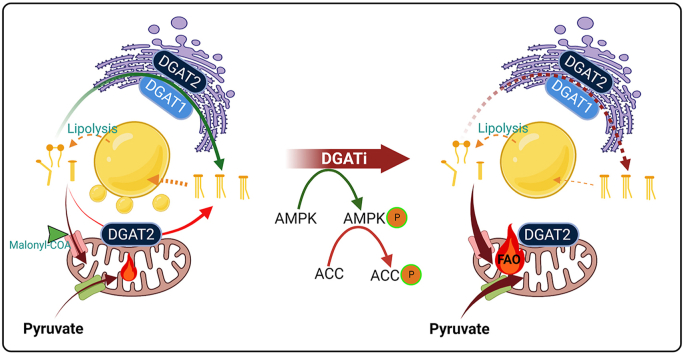

## 1 Introduction

Lipids stored in adipose tissue (i.e., triglycerides (TAG) and other lipid species stored in lipid droplets, LDs) are important reserves for energy and the synthesis of structural lipids [1]. LDs also act as scavenging organelles to quench excess FAs that might otherwise be detrimental to systemic health [1]. Thus, an intricate interplay between lipolysis and esterification (lipogenesis) balances systemic lipid homeostasis. Emerging findings suggest that in healthy subjects, the activity of this cycle of unstimulated lipolysis and re-esterification seems to correlate with metabolic health [[2], [3], [4], [5]], however, exactly how this cycle impacts overall health remains unknown. Previous studies have suggested that adipocytes re-esterify a considerable fraction of FAs under basal as well as stimulated lipolytic conditions [[6], [7], [8]]. For example, in response to isoproterenol (iso) stimulation, re-esterification is induced as a mechanism to prevent lipotoxicity, rather than to preserve TAG content [6,8].

The last enzymatic reaction which transfers the fatty acyl group to diacylglycerol (DAG) to synthesize TAG is catalyzed by two evolutionarily unrelated enzymes i.e., Diacylglycerol O-acyltransferase 1 or 2 (DGAT1 or DGAT2) [[9], [10], [11]]. The role of DGAT1 has been studied extensively, both genetically and pharmacologically [6,8,9,[12], [13], [14], [15], [16]]*. Dgat1* ablation ameliorates insulin resistance and imparts resistance to diet-induced obesity [17]. Consistently, pharmacological inhibition of DGAT1 improves insulin sensitivity and lipid/glycemic balance [[17], [18], [19]]. On the contrary, partly because of the embryonic lethality of *Dgat2* knockout mice, the physiological understanding of the role of DGAT2 is limited [20]; recent studies provide a unique insight into the specific role of DGAT2 in adipose tissue and the liver [9,21]. DGAT2 is shown to facilitate the use of glucose for energy generation by esterifying the glucose-derived de novo generated FAs to a pool of triglyceride that is rapidly hydrolyzed to generate FAs for mitochondrial FAO in stimulated brown adipocytes [22]. However, these studies focused on the stimulated state where the lipolytic flux is very high. The effect of re-esterification, specifically that of lipolysis derived endogenous FAs, remains unclear to date.

DGAT1 and DGAT2, besides a partial functional overlap, show their substrate specificities [9]. We thus argue that only a combined inhibition (D1+2i) can reveal the true role of re-esterification in energy homeostasis. To avoid adaptive response to gene knockdown or knockout, we used specific pharmacological inhibitors of DGAT1 (PF-04,620,110, D1i) and DGAT2 (PF-06,424,439, D2i) [6,8,15,16,23] for combined DGAT inhibition (D1+2i) in murine adipocytes. We show that D1+2i results in a substantial increase in oxygen consumption caused by lipolysis derived FAs. Different sensitivity to D1+2i at basal versus iso-stimulated conditions modulates energetics without perturbing the transcriptional homeostasis. Further, an enhanced mitochondrial pyruvate import, and activation of AMP-activated protein kinase (AMPK) signaling possibly sustain the diversion of FAs for mitochondrial oxidation. Excess intracellular/extracellular FAs can cause cellular stress and lipo/mitotoxicity, therefore it is crucial to maintain FA levels within a certain range. Our energetic studies and lipolysis flux analysis suggest that re-esterification may not only serve as means to moderate the FA concentration to prevent lipotoxicity, but at the same time to regulate cellular energetics and fuel utilization. Consistently, recent findings that adipose-specific *Dgat1/Dgat2* double knockout mice show substantially higher energy expenditure and reduced RER [24], support the physiological relevance of this pathway.

## 2 Materials and methods

### 2.1 Immortalized brown adipocyte culture differentiation

Murine immortalized (brown) pre-adipocytes (iBA) were a kind gift from the laboratory of Prof. Ronald Kahn [25]. iBA cells were cultured in high glucose DMEM (61,965,026, Gibco) supplemented with 10% FBS in the presence of 1*x* pen-strep antibiotics. The cells were plated on collagenized dishes and the differentiation was induced with an induction cocktail (culture media supplemented with 500 μM IBMX, 1 μM dexamethasone, 20 nM insulin, 1 nM T3, 125 μM at 100% cell confluence. After 48 h, fresh maintenance media (culture media with 20 nM insulin and 1 nM T3) was added. The maintenance media was replaced every other day. Since these cells grow and differentiate as multi-layered cells, the cells were replated on collagen-coated multi-well experiment plates on day five to achieve adipocyte monolayer with optimum cell density.

### 2.2 Primary preadipocyte isolation and differentiation

Cell isolation and differentiation were done as described previously [26]. Male C57/BL6 N mice (five weeks old) were purchased from the Charles River laboratories. After one week of acclimatization in our facility, mice were euthanized with CO_2_ overdose and the whole depots of inguinal WAT or interscapular BAT were collected. From 8 mice, we obtained ∼1600 mg of iWAT and ∼750 mg of iBAT tissue. The adipose tissues were finely minced with scissors and resuspended in collagenase buffer (iWAT in 7 ml buffer, iBAT in 3 ml buffer) with 1 mg/ml collagenase (C6885-1G, Sigma–Aldrich)) and digested for 1 h at 37 °C under agitation. Digested tissue was diluted in an equal volume of culture medium and centrifuged at 300 g for 5 min. The SVF fraction (pellet) was re-suspended in media and passed through a cell strainer (40-μm). Flow-through was then plated on collagen-coated plates. At 100% cell confluency, the differentiation was induced as described earlier [26]. After 48 h, fresh maintenance media (culture media supplemented with 20 nM insulin and 1 nM T3 (for brown cells) or 0.5 μg/ml insulin (for white adipocytes)) was added. The maintenance media was replaced every other day. On day 5, the cells were replated on collagen-coated multi-well experiment plates to achieve adipocyte monolayers at the optimum cell density.

### 2.3 Extracellular flux analysis

**Figure 1:**
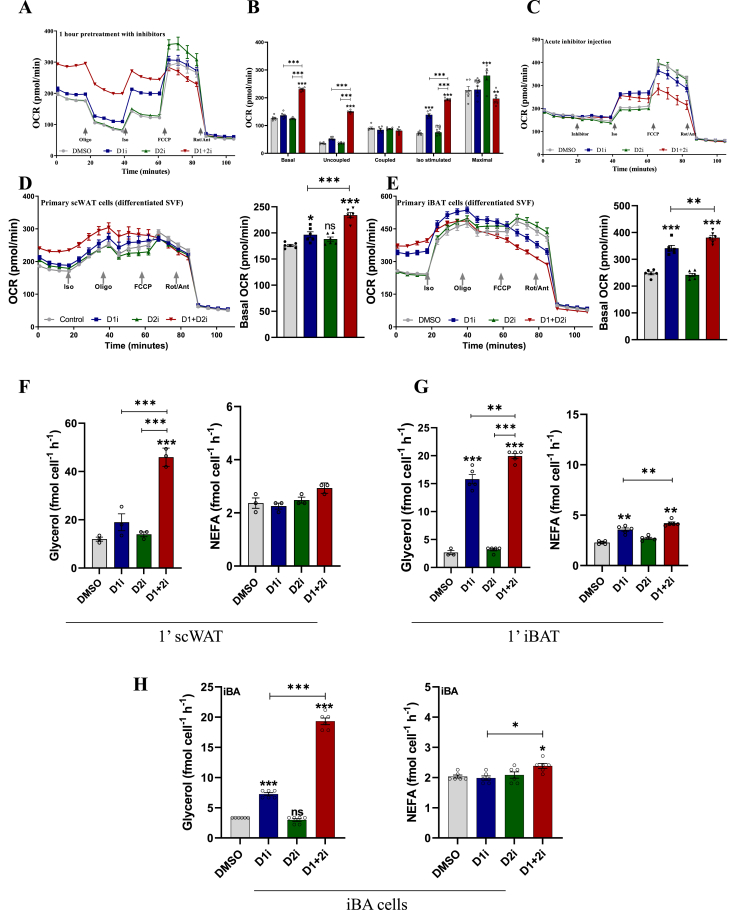
**Inhibition of FA re-esterification enhances OCR**. A-B. OCR measurement and its quantification in the iBA cells pre-treated for 1 h with 2 μ M DGAT inhibitor alone or in combinations (*n* = 6). C. OCR measurement in iBA cells to examine the immediate effects of on-run treatment of 2 μ M DGAT inhibitor alone or in combinations (*n* = 6). D-E. Validation of increase in OCR on *in vitro* differentiated primary iBAT/scWAT cells; bar graph on the right shows AUCs of respective data (*n* = 6). F–H. Lipolytic output analysis in SVF derived primary iWAT cells (*n* = 3) and *in vitro* differentiated primary iBAT cells (*n* = 4), and iBA adipocytes (*n* = 6) respectively. All data are presented as mean ± SEM. one-way ANOVA with Tukey's post hoc test was applied to test the significance of differences. ns: non-significant; ∗*P* < 0.05; ∗∗*P* < 0.01; ∗∗∗*P* < 0.001.

**Figure 2:**
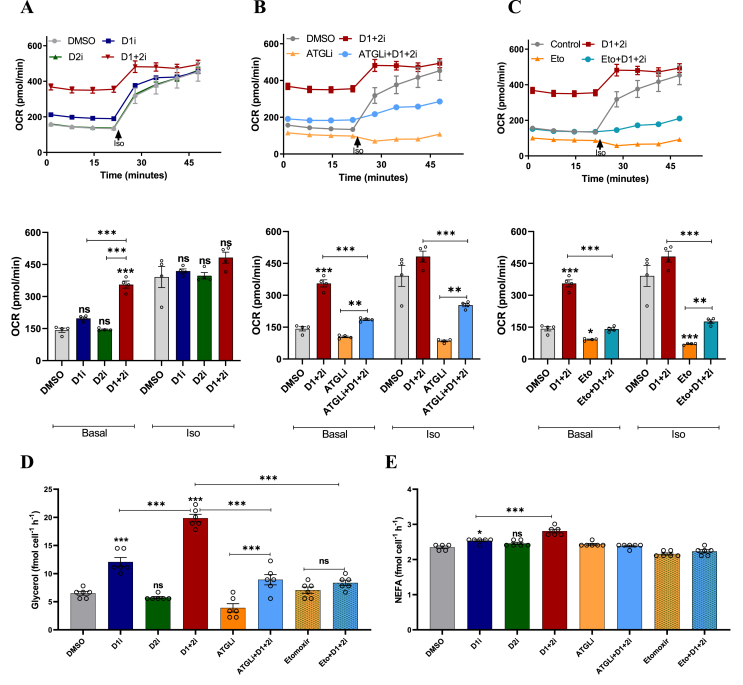
**Lipolysis-derived FAs mediate D1+2i induced OCR**. A-C. Basal and iso-stimulated OCR in the presence of (A) DGAT inhibitors alone, (B) DGAT inhibitors in the presence of the ATGLi (inhibitor of ATGL mediated lipolysis), or (C) DGAT inhibitors in the presence of Etomoxir (inhibitor of CPT1 mediated mitochondrial import of Fatty Acyl-CoA). Respective lower panels show the mean (±SEM) OCR values (*n* = 4). Please note that for better visual comparison, the data for DMSO and D1+2i (from Fig. 2A) are represented in Figure 2B,C as well. D-E. Lipolytic output in iBA in the presence of DGAT inhibitors in combination with ATGLi or Etomoxir. (D) The left panel depicts glycerol release and (E) the panel on the right depicts NEFA levels in the parallel samples collected from the same wells (*n* = 6). All data are presented as mean ± SEM. one-way ANOVA with Tukey's post hoc test was applied to test the significance of differences. ns: non-significant; ∗*P* < 0.05; ∗∗*P* < 0.01; ∗∗∗*P* < 0.001.

On day 5 of differentiation, adipocytes were replated on seahorse XFe96/XF Pro FluxPak cell culture plates at the density of 10,000 cells/well. On the night of day 6, XFe96/XF Pro sensor cartridge was filled with 200 μl Seahorse XF calibrant solution per well and incubated overnight in a CO_2_-free incubator at 37 °C. On day 7, cells were washed 2*x* with seahorse medium (XF assay medium supplemented with 4.5 g/L glucose, 2 mM pyruvate, 2 mM glutamax, pH 7.4). The inhibitors (2 μM) were dissolved in the seahorse medium (with/without 1% BSA) and incubated in a CO_2_-free incubator for 1 h. Meanwhile, the XFe96/XF Pro sensor cartridge ports were filled with various treatment solutions to be injected and the cartridge was kept in the XFe96 analyzer for equilibration followed by the readout. For the experiment in Figure 1A, the basal oxygen consumption rate (OCR) was measured followed by sequential injection of Oligomycin (1 μg/ml), iso (1 μM), FCCP (1 μg/ml), and Ant/Rot combination (2 μg/ml). For the experiment in Figure 1C, no inhibitor pre-treatment was performed. After baseline OCR measurement, 2 μM DGAT inhibitors (or DMSO), iso, FCCP, and Ant/Rot combination were injected sequentially. For the experiment in Figure 1D–E, cells were pre-treated for 1 h with the inhibitors as for Figure 1A and basal OCR was measured followed by mentioned injections. For the experiment in Figure 2A–C, the cells were pre-treated for 1 h with respective inhibitor combinations in a CO_2_-free incubator. After basal OCR measurement, iso-stimulated OCR was recorded.

To calculate various respiratory parameters from the seahorse experiment (Figure 1A,B), non-mitochondrial respiration value (OCR after Rot/Ant injection) was subtracted from all readings to get mitoOCR. All parameters are based on this mitoOCR (e.g., ATP-coupled respiration is the drop in mitoOCR after oligomycin injection; iso-stimulated OCR is the change in mitoOCR after iso injection, etc.). Wherever basal OCR is presented as a bar graph (Figure 1, Figure 2), the average basal OCR of all replicates is taken.

### 2.4 Lipolysis assay

On day 5 of differentiation, iBA adipocytes were replated on collagen-coated 96 well plates. On day 7, the inhibitor/combinations were dissolved in phenol red-free DMEM (Gibco) and added to washed cells in the presence or absence of 1 μM iso. After a 2-hour incubation in a CO_2_-free incubator, 40 μl media was collected in two transparent 96 well plates, one each for non-esterified FA (NEFA) and glycerol. Extracellular glycerol was measured using the Glycerol reagent (Sigma–Aldrich) and reading absorbance at 540 nm on Synergy MX/Gen5 software (BioTek). NEFA levels were measured by NEFA-HR kit (Wako) using R1/R2 reagents. The concentrations were calculated by using the absorbance from glycerol (G7793, Sigma) or NEFA (276–76491, Wako) standard. For intracellular NEFA/glycerol measurement, cells were lysed in 50 μl of RIPA lysis solution followed by the addition of 50 μl 2*x* TBS (with 1% BSA), and 40 μl sample was collected in two transparent 96 well plates, one each for NEFA and glycerol.

### 2.5 qRT-PCR

Differentiated brown adipocytes were plated on 24 well plates. Cells were treated with the inhibitor combinations for 2 h. The RNA isolation was performed using the standard Trizol chloroform separation method and was digested with DNase to remove genomic DNA. A total of 1 μg RNA was converted into cDNA using a high-capacity cDNA synthesis kit (4,368,814, Applied Biosystems). SYBR green reagent-based qRT-PCR was performed on a ViiA 7 Real-Time instrument (Applied Biosystems) and the data were analysed using ΔΔC_T_ method. The primer sequences are provided in Table 1.

**Table 1:** List of the primer sequences used in this study.

Gene	Forward primer (5' -> 3′)	Reverse primer (5' -> 3′)
*Aqp7*	ATGAGGCATTCGTGACTGGG	CCCCAAGGACGGTAACAAGG
*Atf4*	CCTGAACAGCGAAGTGTTGG	TGGAGAACCCATGAGGTTTCAA
*Atf5*	TGGGCTGGCTCGTAGACTAT	GTCATCCAATCAGAGAAGCCG
*Atgl*	CTGAGAATCACCATTCCCACATC	CACAGCATGTAAGGGGGAGA
*Catalase*	GGAGGCGGGAACCCAATAG	GTGTGCCATCTCGTCAGTGAA
*Dgat1*	AACCGAGACACCATAGACTACT	CTTCAGGGTGACTGCGTTCTT
*Dgat2*	TAGAAGAGGACGAGGTGCGA	GTCTTTGTCCCGGGTATGGG
*GyK*	GTCAGCAACCAGAGGGAAACC	CCACGGCATTATAGAGAGGCT
*Hsp60*	CACAGTCCTTCGCCAGATGAG	CTACACCTTGAAGCATTAAGGCT
*Hsp70*	TGGTGCAGTCCGACATGAAG	GCTGAGAGTCGTTGAAGTAGGC
*Lc3*	TTATAGAGCGATACAAGGGGGAG	CGCCGTCTGATTATCTTGATGAG
*Nrf1*	TATGGCGGAAGTAATGAAAGACG	CAACGTAAGCTCTGCCTTGTT
*Parkin1*	TCTTCCAGTGTAACCACCGTC	GGCAGGGAGTAGCCAAGTT
*Pgc1α*	CTCTCAGTAAGGGGCTGGTTG	CGAATGACGCCAGTCAAGC
*Pink1*	TTCTTCCGCCAGTCGGTAG	CTGCTTCTCCTCGATCAGCC
*Pparg*	GTGGGGATAAAGCATCAGGC	CCGGCAGTTAAGATCACACCTA
*Sod1*	AACCAGTTGTGTTGTCAGGAC	CCACCATGTTTCTTAGAGTGAGG
*Sod2*	CAGACCTGCCTTACGACTATGG	CTCGGTGGCGTTGAGATTGTT

### 2.6 Confocal microscopy and operetta

On day 5 of differentiation, iBA adipocytes were replated on collagen-coated 96 well μClear black-wall, transparent bottom plates. On day 7, the inhibitor/combinations were dissolved in phenol red-free DMEM (Gibco) and added to cells in the presence or absence of 1 μM iso. After a 2-hour incubation at 37 °C, cells were fixed for 15 min at room temperature with 4% paraformaldehyde. For confocal microscopy, fixed cells were permeabilized with 0.1% Triton ×100 dissolved in PBS. Nonspecific epitopes were blocked in 2% BSA containing PBST (PBS + 0.1% tween 20). Next, cells were incubated with an anti-ATP5I antibody (1:300 in 2% BSA-PBST) at 4 °C overnight. The next day, cells were washed 3 times with PBST and incubated with Alexa flour 568 anti-rabbit antibody (1:500 in 2% BSA-PBST) for 1 h. Cells were then washed three times with PBST and then incubated with PBST containing 1:2000 lipidtox red and 1 μM Hoescht. After 30 min, the cells were washed two times with PBST and kept in PBS and were imaged on Olympus FluoView 3000 confocal microscope.

For operetta analysis of LDs, fixed cells were washed three times with PBST and incubated with PBST containing 1:2000 LD540, 1:1000 Syto60, and 1 μM Hoescht. After 30 min, the cells were washed three times with PBST and kept in PBS and were imaged on an operetta high content analysis system (PerkinElmer). Each group included 6 wells; from each well, 12 fields (with 300–400 cells per field) were analyzed and the well average was used as one replicate. The data was analyzed using harmony software.

*BODIPY™ FL C*_*16*_*localization*: On day 5 of differentiation, iBA adipocytes were replated onto collagen-coated coverslips. On day 7, cells were treated with 100 μM BODIPY™ FL C16 for 12 h in complete DMEM. Next, cells were washed, and fresh media was added. Following a 6-hour chase period to get rid of free BODIPY™ FL C16 (Thermo Fisher, D3821), cells were washed two times and treated with 5 μM D1+2i or equivalent DMSO in serum-free DMEM (containing 0.1% BSA). After 30 min, 100 μM MitoTracker Deep Red FM (Invitrogen, M22426) was added on top. After another 30 min incubation, cells were washed three times with PBS and fixed in 4% paraformaldehyde followed by mounting onto slides with Fluoromount-G mounting medium, with DAPI (Invitrogen, 00-4959-52). Slides were scanned on Olympus FluoView 3000 confocal microscope.

*NBD-palmitoyl-CoA incorporation*: Cells were replated as described above. On day 7, cells were treated with 5 μM D1+2i or equivalent DMSO in serum-free DMEM (containing 0.1% BSA). After 30 min, 100 μM NBD-palmitoyl-CoA was added along with 100 μM MitoTracker Deep Red FM. After 30 min incubation, cells were washed three times with PBS and mounted onto slides with Fluoromount-G mounting medium, with DAPI (Invitrogen, 00-4959-52). Slides were scanned on Olympus FluoView 3000 confocal microscope.

*Microscopy image analysis*: Colocalization was measured by using the JaCoP plugin in Image J software to get Pearson coefficient or mander's colocalization coefficient M (fraction of BODIPY™ FL C_16_ colocalizing with MitoTracker). The intensity distribution profile was generated using the Graphics plugin in Image J. The intensity of NBD-palmitoyl-CoA was quantified by using image J and data were plotted in Graph Pad Prism 9.2.0.

### 2.7 ROS assay

On day 5 of differentiation, cells were replated onto the collagen-coated clear bottom, black wall μClear plates. On day 7, cells were washed three times and treated with DMSO or D1+2i for 90 min followed by the addition of ROS reagent as recommended by the kit (ab139476 ROS/Superoxide Detection Assay Kit) and were read on a microplate reader at Ex = 488 nm, Em = 520 nm.

### 2.8 JC1 aggregation assay

On day 5 of differentiation, cells were replated μClear plates as described above. On day 7, cells were washed three times and treated with DMSO or D1+2i for 90 min followed by the addition of 200 nM JC1 dye (ab113850 JC-1). After 20 min, plates were washed three times with PBS and were read on a microplate reader at Ex = 535 nm, Em = 595 nm.

### 2.9 ^13^C-palmitate tracing and endogenous lipidomic analysis

^13^C-Palmitate was conjugated to BSA to a stoichiometry of 1:3 BSA: palmitate. A total of 1.8 mg ^13^C-palmitate was dissolved in 50 μl ethanol then dropwise added to BSA solution (in phenol red-free DMEM) and stirred at 900 rpm for 1 h at 37 °C. The BSA-palmitate conjugate was added to pre-washed cells to a final concentration of 20 μM ^13^C-palmitate. Isotope negative control (INC) cells received an equal amount of ^12^C palmitate and the ^13^C enrichment value of INC was subtracted from all experimental groups to normalize for the background. After 2 h, the cells were washed twice with cold PBS. Next, 1-ml of ice-cold methanol: isopropanol 1:1 (v/v) was added, and the plates were shaken manually for 30 s. The plates were incubated at −80 °C for 5 min followed by the collection of cells by scaping. The samples were snap-frozen in liquid nitrogen and stored at −80 °C until processed. For analysis, the samples were centrifuged at 16000 g for 10 min and the supernatant was transferred to a new tube, and dried by speed vac under N_2_. The dried lipid fraction was dissolved in 50 μl methanol: isopropanol (1:1) by vortex and incubation in a thermomixer for 10 min. The solubilized lipid fraction was centrifuged at 16000 g for 10 min and the supernatant was transferred to glass LC/MS vials. The lipids were separated on C18 reverse phase chromatography (Acquity BEH 100 mm column (Waters) with 2.1 mm internal diameter and 1.7 μm particle diameter) attached to a Vanquish LC pump (Thermo Fisher Scientific) with the following mobile phases: (i) acetonitrile: water (6:4) with 10 mM ammonium acetate and 0.1% formic acid, (ii) isopropanol: acetonitrile (9:1) with 10 mM ammonium acetate and 0.1% formic acid [27,28]. The following gradient (0.6 ml/min) was used: 0.0–2.0 min (isocratic 30% B), 2.0–2.5 min (ramp 30–48% B), 2.5–11 min (ramp 48–82% B),11–11.5 min (ramp 82–99%), 11.5–12 min (isocratic 100% B), 12.0–12.1 min (ramp 100-30% B) and 12.1–15 min (isocratic 30% B).

The liquid chromatography was coupled to a hybrid quadrupole-orbitrap mass spectrometer (Q-Exactive HFx, Thermo Fisher Scientific). A Full scan acquisition in negative and positive ESI was used to scan 200–2000 *m*/*z* at a resolution of 120,000 and AGC target of 1e6, max injection time of 200 ms. Data-dependent scans (top 10) were acquired using normalized collision energies (NCE) of 20, 30, and 50, at a resolution of 15,000, and an AGC target of 1e5.

Identification of the specific lipids was achieved using four criteria [1]: high accuracy (*m*/*z* within 5 ppm shift from the predicted mass) and high resolution (resolving power 70,000 at 200 *m*/*z*) [2], isotopic pattern fitting to expected isotopic distribution [3], comparing the retention time to an in-house database, and [4] the fragmentation pattern matching to an in-house experimentally validated lipid fragmentation database. All the isotopologue peaks were quantified, the mass isotopomer distributions (MDVs) were calculated, and the fractional labeling was calculated as described earlier [29] using Compound Discoverer 3.1 (Thermo Fisher Scientific).

For the lipidomic analysis of endogenous lipids, cells were treated with inhibitor combinations in the presence/absence of BSA for 2 h. After 2 washes with ice-cold PBS, 0.5 ml methanol: isopropanol 1:1 (v/v) containing 2 μl/ml SPLASH internal control standard mix (Avanti, 330,707-1 EA) was added. Following a 5-minute incubation at −80 °C, total lipids were carefully extracted and transferred to Eppendorf tubes. Sample processing and data analysis were performed as described above for ^13^C palmitate tracer experiments.

### 2.10 Substrate specificity test

On day 5 of differentiation, iBA cells were replated on seahorse cell culture plates. On day 7, the cells were washed 3*x* with assay medium (XF base media supplanting with 2 mM Glutamax, 1 mM sodium pyruvate, and 10 mM glucose, and pH was brought to 7.4 with NaOH). Cells were incubated with DMSO or D1+2i in 150 μl assay media and incubated in a CO_2_-free incubator at 37 °C. After basal OCR measurement, BPTES (6 μM) or UK5099 (2 μM) or a combination of either of the compound with Etomoxir (100 μM) was injected followed by OCR measurements. The OCR values of the four time points were averaged for statistical analysis using one-way ANOVA.

### 2.11 AdipoRed total lipid assay

On day 5 of differentiation, cells were replated μClear plates as described above. On day 7, cells were washed three times and treated with given inhibitor combinations for 2 h. Cells were then washed two times and incubated with 100 μl (40*x* dilution in PBS) adipoRed assay reagent (Lonza PT 7009) for 15 min and read on a microplate reader using Ex = 485 nm, Em = 535 nm.

### 2.12 Western blotting

A total of 20 μg of protein was loaded on 12% SDS-PAGE gel. After electrophoresis and transfer of protein onto nitrocellulose membrane, the membrane was blocked in 5% BSA in TBST followed by overnight incubation at 4 °C with primary antibody (anti-AMPKα: Rabbit mAb #5831 from CST; anti-pAMPKα: Rabbit mAb #2535 from CST; anti-HSP90: Rabbit mAb #4877 from CST; anti-ACC1: #4190 from CST; total anti-pACC (Ser79): Rabbit mAb #11818 from CST) in TBST containing 5% BSA. After four washes with PBST, the membrane was incubated with HRP-conjugated goat anti-rabbit secondary antibody (EMD Millipore #401393-2 ml). After 1 h of incubation at room temperature, the membrane was washed 4*x* with TBST and a chemiluminescent blot was developed on the ImageQuant system (LAS 4000 mini, GE Healthcare). Band intensity was quantified using Image lab 6 (BioRad laboratories).

### 2.13 Statistical analysis

All data are presented as mean ± SEM. Two group comparisons were tested for significance using a two-tailed unpaired Student's t-test. Multiple group comparisons were performed by one-way ANOVA. All statistical analyses were performed using GraphPad Prism 9. Statistical differences are indicated ∗ for P < 0.05, ∗∗ P < 0.01, and ∗∗∗ P < 0.001.

## 3 Results

### 3.1 Inhibition of FA re-esterification induces OCR

Although the magnitude may vary in a context-dependent manner (influenced by energetic status, experimental setup, and physiological state of the cells), the fate of the majority of lipolytic FAs in adipocytes seem to be re-esterification to TAG [[6], [7], [8]]. Therefore, inhibition of re-esterification should lead to increased FA levels, however, the fate of excess FAs/FA-CoA upon inhibition of re-esterification (D1+2i) is unknown. We, therefore, tested an acute inhibition of DGAT1/2 and measured the oxygen consumption rate in iBAs. We first optimized the inhibitor doses and incubation times and found that 2 μM inhibitors treated for 1 h before seahorse assay is optimum (Fig. S1A, B). At the basal level, D1i caused a mild increase in oxygen consumption rate (OCR) while D2i showed no effect. A combined inhibition (D1+2i, 2 μM each) led to a substantial increase in OCR at basal conditions (Figure 1A,B). A similar trend was observed after iso-stimulation, albeit with a more pronounced effect of D1i (Figure 1A,B). Noticeably, a large fraction of increased OCR was attributed to uncoupled respiration, conceivably due to the uncoupling capacity of brown adipocytes. We next tested if OCR increases acutely after DGAT inhibition or follows a lag phase, which would imply that OCR increase is a secondary, adaptive response. In contrast to 1-hour pre-treatment, in-run injection of inhibitors caused only a mild increase in basal OCR while iso-stimulated OCR was higher in D1i or D1+2i (Fig. 1C). Nonetheless, the extent of increase in OCR was substantially lower compared to 1-hour pre-treatment, suggesting either a cumulative effect or an adaptive intermediate response, which amplifies/sustains the increase in OCR, particularly at the basal state.

We further validated the findings in primary adipocytes from *in vitro* differentiated murine stromal vascular fraction (SVF) from inguinal brown adipose tissue (iBAT) or inguinal subcutaneous white adipose tissue (scWAT). Since we wanted to compare the total basal and total iso-stimulated OCR, we injected iso after basal readings followed by oligomycin. Although tissue-specific difference in sensitivity towards D1i is likely, the D1+2i-induced increase in OCR was consistently higher (Figure 1D,E). We next examined if D1+2i modulates lipolytic flux by measuring the release of NEFA and glycerol in the culture media. In SVF-derived primary scWAT adipocytes D1i but not D2i led to a mild insignificant increase in glycerol release (Fig. 1F). Interestingly, in line with the OCR data, D1+2i led to a ∼4.5-fold increase in the glycerol levels while the FA levels remained comparable across all groups, suggesting that the excess FAs arising from enhanced lipolysis are possibly used in mitochondrial to drive OCR (Fig. 1F). Consistently, *in vitro* differentiated primary iBAT adipocytes exhibited a ∼6-fold increase in glycerol release upon D1+2i (Fig. 1G). It is noteworthy that compared to changes in the OCR, primary iBAT adipocytes were more responsive to D1i than the immortalized brown adipocytes. Following scWAT cells or primary iBAT cells, iBA adipocytes also showed a similar trend of glycerol/FA release upon D1+2i (Fig. 1H). To confirm that the OCR phenotype is due to inhibition of re-esterification and is not a DGATi-specific effect, we used MGAT2 inhibitor which induced a very slight change in OCR while a combination of D1+2i and MGAT2i further increased the OCR in an additive manner (Fig. SI1C). An MGAT3 inhibitor (MGAT3 is a pseudogene in mice) was used as a control and did not affect OCR (Fig. SI1D). These data suggest a balancing of re-esterification between lipolysis and FAO and that the inhibition of re-esterification possibly channels activated fatty acids to mitochondria leading to an increase in OCR.

### 3.2 Lipolysis-derived FAs enter mitochondria to induce OCR upon D1+2i

To further examine if mitochondrial FAs mediate the increased OCR we blocked either lipolysis (ATGLi) or mitochondrial import of FAs (Etomoxir) in combination with DGATi. Inhibition of either DGAT isoforms alone or in combination showed the same basal effect as described before (Fig. 2A). The inhibition of lipolysis by ATGLi resulted in a reduction in OCR at the basal level and blunted iso-stimulated increase in OCR (Fig. 2B). Also, the D1+2i effect was dampened by ATGLi (Fig. 2B) suggesting that lipolysis derived FAs are likely mediators of the increased OCR. The slight increase in OCR observed in ATGLi + D1+2i compared to ATGLi alone could arise from residual ATGL activity or ATGL-independent HSL-mediated lipolysis. Similarly, blockade of mitochondrial import of activated FAs by the CPT1-inhibition largely abolished the effect of D1+2i in the basal as well as iso-stimulated state (Fig. 2C). It should be noted that DGAT inhibition in combination with ATGLi or etomoxir did not reduce OCR below basal levels, indicating a remnant housekeeping activity. These results together suggest that the D1+2i-induced OCR is mediated by an increased mitochondrial influx of lipolysis-derived FAs.

Quantification of lipolysis (glycerol/FA release) further supported the lipolytic contribution to the D1+2i induced OCR (Figure 2D,E). D1i led to a ∼2-fold increase in glycerol release, while D1+2i led to a ∼3-fold increase. Given that the D2i by itself showed no effect, a compensatory contribution could be envisaged. One possibility is that the increase in OCR is mediated by a pool of FAs that are redundantly used by DGAT1 as well as DGAT2 and thus only a combined inhibition (i.e., D1+2i) leads to a large increase in OCR. We also examined the role of ATGLi and etomoxir on lipolysis. ATGLi inhibited the basal glycerol release and blunted the D1+2i-induced increase. Etomoxir treatment also suppressed the D1+2i-induced glycerol release. To rule out the cellular retention of FAs (as NEFA or acyl-CoA) upon D1+2i, we analyzed intracellular FA and glycerol content (Fig. SI2). We did not observe intracellular accumulation of FA in D1+2i treated cells, pointing towards active utilization of excess FAs. These results suggest that the FAs generated from lipolysis are utilized to fuel OCR (Figure 1, Figure 2). The data also imply that the rate of re-esterification by DGATs likely modulates the mode and extent of mitochondrial partitioning of FAs.

### 3.3 Differential utilization of exogenous 13C-palmitate upon D1+2i

**Figure 3:**
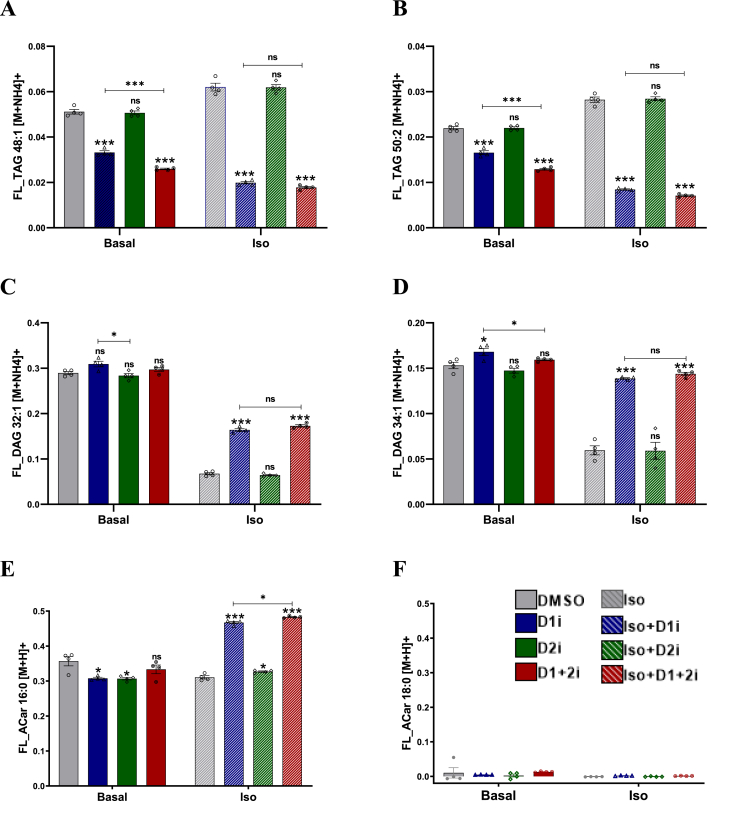
**Differential utilization of exogenous**^**13**^**C palmitate upon D1+2i**. A-F. Fraction ^13^C labelling (%) of FA derivatives upon incubation of iBA adipocytes with ^13^C Palmitate. Cells (*n* = 4) were incubated with ^13^C Palmitate for 2 h followed by extraction of metabolites and measurement of fractional labelling of (A) TAG 48:1 [M + NH4]+; (B) TAG 50:2 [M + NH4]+; (C) DAG 32:1 [M + NH4]+; (D) DAG 34:1 [M + NH4]+; (E) ACar 16:0 [M+H]+; (F) ACar 18:1 [M+H]+. All data are presented as mean ± SEM. one-way ANOVA with Tukey's post hoc test was applied to test the significance of differences. ns: non-significant; ∗*P* < 0.05; ∗∗*P* < 0.01; ∗∗∗*P* < 0.001.

To better understand the mechanism of mitochondrial diversion of FAs, we considered two possibilities: (i) the excess FA-CoAs that were otherwise destined for re-esterification are directly diverted to mitochondria for FAO, or (ii) an adaptive change in energetic signaling mediates the increase in OCR. Therefore, we measured the activation of exogenous ^13^C-palmitate into acylcarnitine and its direct incorporation into prominent glyceride species (Figure 3). Due to the role of DGATs in esterifying FA to TAGs, we first assessed the incorporation of ^13^C-palmitate into TAG species (Figure 3A,B). At the basal level, incorporation of ^13^C-palmitate into 48:1 TAG was inhibited by D1i while D2i showed no effect. Consistent with other results, D1+2i further decreased the label incorporation than D1i alone (Fig. 3A). Under iso-stimulated conditions, the incorporation of ^13^C-palmitate to 48:1 TAG was enhanced compared to basal control. However, the effect of D1i was so pronounced that the total ^13^C-palmitate incorporation into 48:1 TAG was even lower than the D1i at the basal level. Interestingly, under the stimulated conditions, DGAT1 appears to be the sole esterifying enzyme as the D1+2i was comparable to D1i (Fig. 3A). These observations hold true also for 50:2 TAG (Fig. 3B). The basal incorporation of ^13^C-palmitate to DAG (30:1 as well as 34:1) was largely unaffected by DGATi (Figure 3C,D). However, upon iso-stimulation, the incorporation of ^13^C-palmitate to DAG species in the control group was reduced significantly (Figure 3C,D), possibly due to enhanced cycling. D1i (and D1+2i) led to an increased accumulation of ^13^C-palmitate DAG as the third acylation reaction was inhibited by DGATi. This could also be due to the availability of excess fatty acyl-CoA due to D1i and suggest the existence of a crosstalk between monoacyl glycerol acyl transferases and DGAT action. These findings demonstrate that: (i) there is a significant re-esterification of FA to TAG/DAG at the basal as well as under the stimulated conditions, (ii) although under iso-stimulated conditions DGAT1 is the major re-esterifying enzyme, under basal conditions, DGAT2 can actively esterify FAs and thus only a combined inhibition results in maximal suppression of DGAT action.

One of the most striking observations was the changes in ^13^C-palmitoyl-carnitine (Acyl carnitine, 16:0) levels (Fig. 3E). At basal levels, D1i or D2i led to a small decrease in ^13^C-palmitoylcarnitine when compared to the control group. Surprisingly, the D1+2i did not alter the ^13^C-palmitoylcarnitine generation. Given the dependence of OCR on mitochondrial uptake of lipolytic FAs, the unchanged incorporation of ^13^C-palmitate to ^13^C-palmitoylcarnitine in DGAT1+2 inhibited cells at basal conditions is difficult to explain. Therefore, we analyzed the total acylcarnitine content (M+0, M+16 or the sum ([M+0] and [M+16])) which was substantially higher in the D1+2i than in the control. Fractional labeling represents the fraction of the total palmitoylcarnitine pool with a ^13^C isotope (not the absolute quantity). The results suggested that upon D1+2i, ^13^C palmitoylcarnitine increases but a proportional increase in endogenous (unlabelled) palmitoylcarnitine renders the fractional labeling unchanged despite increased absolute values. A similar phenomenon seems to be responsible for the unchanged DAG levels at basal conditions (Fig. SI3A-C). A comparable level of the sum of isotopologues in controls (with ^13^C-palmitate versus without ^13^C-palmitate) demonstrates the consistency of the data (Fig. SI3D). In contrast to basal conditions, under iso-stimulated condition, D1i led to a ∼30% increase in total ^13^C-palmitoylcarnitine while D2i caused a small but significant increase in ^13^C-palmitoylcarnitine levels (Fig. 3E). Combined D1+2i inhibition elicited an additive effect on the accumulation of acylcarnitine. The non-labeled acylcarnitine (18:0) was minimally affected (Fig. 3F). These results suggest a role of re-esterification for a differential utilization of FAs based on the abundance in basal and iso-stimulated conditions.

### 3.4 Acute inhibition of re-esterification causes a selective shift in fuel utilization

**Figure 4:**
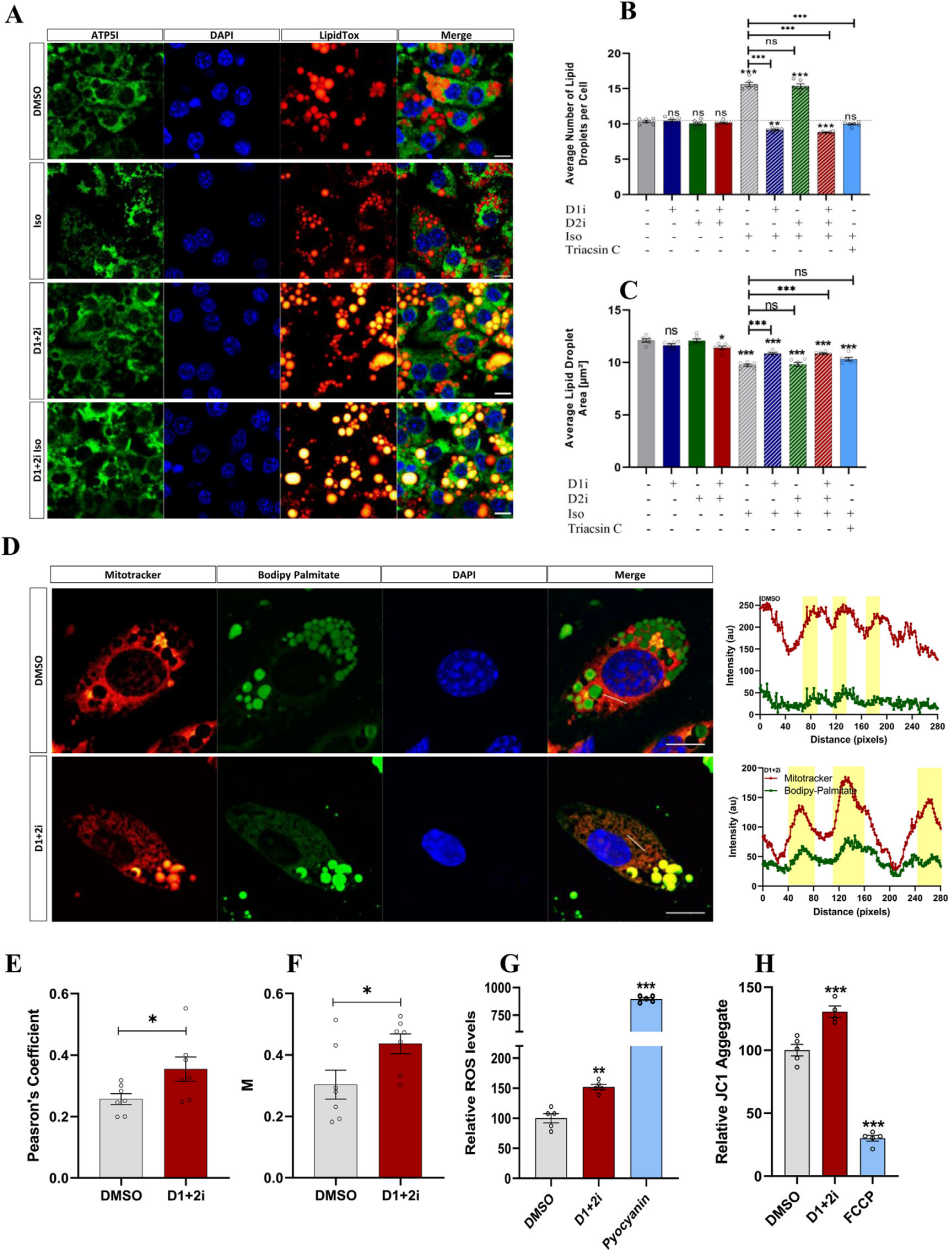
**DGATi induced changes in LD distribution and FA mobilization to mitochondrial**. A. Confocal images of iBA cells stained for mitochondria (ATP5I, green), LDs (Lipidtox, red), and nucleus (Hoechst, blue). Cells were treated for 2 h with vehicle or D1+2i in the absence and presence of iso and then were fixed and stained. Scale bar, 10 μm. B–C. Quantification of (B) average number of LDs per cell, and (C) average LD area. In a parallel experiment to confocal imaging (A), cells were processed for operetta analysis, and the data were analyzed using harmony software provided by the vendor. (n = 6 wells per group). Each well value represents an average of 12 fields. D-F. Confocal images of iBA showing localization of Bodipy-palmitate to LDs/mitochondria. (D) iBA adipocytes were treated overnight with Bodipy-palmitate in complete media. Next day, cells were washed, and cells were incubated in Bodipy-palmitate free media for 6-hour. Cells were then washed and treated with DMSO or D1+2i for 1 h followed by cell fixation and visualization. The panel on the right shows the intensity profile of Mitotracker or Bodipy-palmitate in given linear ROI (red line in merge channel). Scale bar, 10 μm. (E) Pearson's correlation coefficient, and (F) Mander's colocalization coefficient (fraction of bodipy-palmitate overlapping with mitotracker red) (*n* = 7). G. Reltive ROS levels in iBA cells measured after 2 h of treatments (*n* = 5). H. JC1 staining of the iBA cells showing increased JC1 aggregates as a function of increased mitochondrial potentilal upon respective treatments (*n* = 5). All data are presented as mean ± SEM. For the data presented in graph E and F, two tailed t-test was applied while for other data one-way ANOVA with Tukey's post hoc test was applied to test the significance of differences. ns: non-significant; ∗*P* < 0.05; ∗∗*P* < 0.01; ∗∗∗*P* < 0.001.

Based on our observation of a shift in FA utilization upon acute D1+2i, we wondered if inhibition of re-esterification causes any global/long-lasting alteration in immortalized adipocytes. Therefore, we tested if D1+2i leads to localized changes in lipid droplet (LD) morphology and/or number. ATP5I staining was used to label the mitochondria. Although ATP5I expression (and that of other representative respiratory complex proteins along with ECSH1) was not significantly different between the groups, we observed marginally increased peri-LD mitochondria in D1+2i treated cells (Figure 4A; Figure SI4; Fig SI5A). We observed a negligible reduction in the total number of LDs upon D1+2i at the basal level, likely due to the low LD turnover rate (Figure 4A,B). The iso-stimulation of control cells led to the appearance of multiple smaller droplets (Figure 4A,B). However, the new small LD appearance was abolished in D1i or D1+2i, but not in D2i. Consistently, Triacsin C treatment, which blocks FA activation, also inhibited the appearance of new small LDs during iso-stimulation (Fig. 4B). These results suggest that DGAT1 is the major re-esterifying enzyme under conditions of iso-stimulated lipolysis. Moreover, under basal conditions, an increase in lipolysis enhances the FA availability without affecting overall LD morphology in the timeframe used here. Besides the LD number, the changes in the size of LDs also reflect consistent and complementary features. Upon D1i or D1+2i, there was a slight reduction in the LD area, possibly because LD-derived FAs are used to drive OCR. Consistently, upon iso-stimulation, all groups displayed significantly smaller LDs (Fig. 4C). Interestingly, however, the average size of LDs in the iso-stimulated D1i or D1+2i group appears to be larger than that of stimulated control cells (Figure 4C; Fig. SI4). To test if under iso-stimulated conditions D1i and D1+2i block recruitment of new LDs whereby the remaining large LD would skew the size estimation, we blocked FA activation by triacsin C, which resulted in a similar phenotype (Fig. 4C).

We next examined if inhibition of re-esterification also alters the gene expression of lipid metabolism-related genes or the genes involved in mitochondrial health. At basal conditions, iBA adipocytes showed minimal transcriptional response to D1i, D2i, or even D1+2i (Fig. SI5B). Under iso-stimulated conditions, D1+2i led to a marginally decreased expression of several lipid storage genes (*Pparg, Gyk, Dgat1, Dgat2*), whereas a few other genes involved in mitochondrial homeostasis were down-regulated (*Sod1, Sod2, Cat, Hsp60*). The mitochondrial chaperon, *Hsp70*, was slightly upregulated upon D1+2i (Fig. SI5C). D1+2i led to the upregulation of *Parkin* in the iso-stimulated states. We also tested the expression of *Ucp1* and other metabolically relevant genes (Fig. SI5D,E). We found that iso-treatment itself led to an upregulation of *Ucp1, Pck1, and Fgf21*, and slight downregulation of *Pdk1*. DGATi did not change *Ucp1* expression at basal levels, while in the iso-stimulated state, DGATi caused a slight downregulation of *Ucp1*. In contrast, *Pck1* expression seems to be mainly affected by D1i. Overall, these data suggest that at the tested time points there is only a minimal effect of inhibition of re-esterification at the expression of lipid metabolizing genes and mitochondrial homeostatic genes, rather it selectively impacts LD dynamics and mitochondrial respiration.

We next examined if the lipolysis-derived FAs are channeled to mitochondria by monitoring the mobilization of fluorescent FA (Bodipy Palmitate) from LDs to mitochondria. In control cells, the bodipy signal occasionally colocalized with mitotracker, D1+2i increased the colocalization of bodipy and mitotracker (Figure 4D–F, intensity distribution profile on the right). The mitochondria also seem to reorganize with a higher peri-droplet mitochondrial accumulation observed along with mitochondria infiltration of some partly shreded LDs with the rough, irregular surface (Fig. 4D). In contrast to prelabelled LDs containing bodipy-palmitate, a coincubation of NBD-palmitoyl-CoA with D1+2i showed that D1+2i almost completely blocked the esterification of palmitoyl-CoA into LDs yet some mitochondria showed increased peri-droplet accumulation and increased number of aggregates (Fig. SI5F). It is well established that brown adipocytes generate ROS as a by-product of electron flow across the electron transport chain [30,31]. Two hours of D1+2i treatment caused a mild increase in ROS levels (Fig. 4G). It should be noted that the D1+2i-induced ROS levels are substantially lower than pyocyanin, which was used as a positive control. Furthermore, there was a similar increase in the JC1 aggregate formation (Fig. 4H), an indicator of increased mitochondrial membrane potential. Overall, these results demonstrate that D1+2i increases the mitochondrial channeling of FAs to sustain cellular energy homeostasis.

### 3.5 D1+2i modulates DAG and acylcarnitine levels while other abundant lipids are minimally perturbed

**Figure 5:**
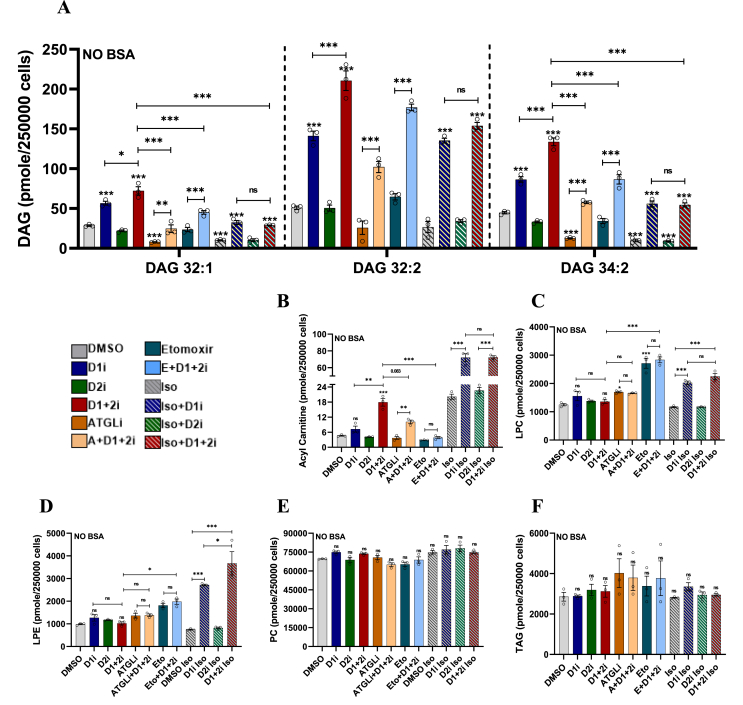
**DGAT inhibition causes selective changes in cellular lipids**. A-F. Lipidomic analysis of differentiated iBA adipocytes treated with different pharmacological combination for 2 h demonstrate (A) increased basal DAG lipids after D1+2i when compared to D1i. In the iso-stimulated state, DGAT1 is the main DGAT as the D1i is as effective as D1+2i. A similar trend is seen for (B) acylcarnitines suggesting diversion of FAs to mitochondria. Other lipid species, (C) LPC, (D) LPE, (E) PC, and (F) TAG showed less prominent changes. Data are presented as mean ± SEM (*n* = 3). A one-way ANOVA with Tukey's post hoc test was applied to test the significance of differences. ns: non-significant; ∗*P* < 0.05; ∗∗*P* < 0.01; ∗∗∗*P* < 0.001.

**Figure 6:**
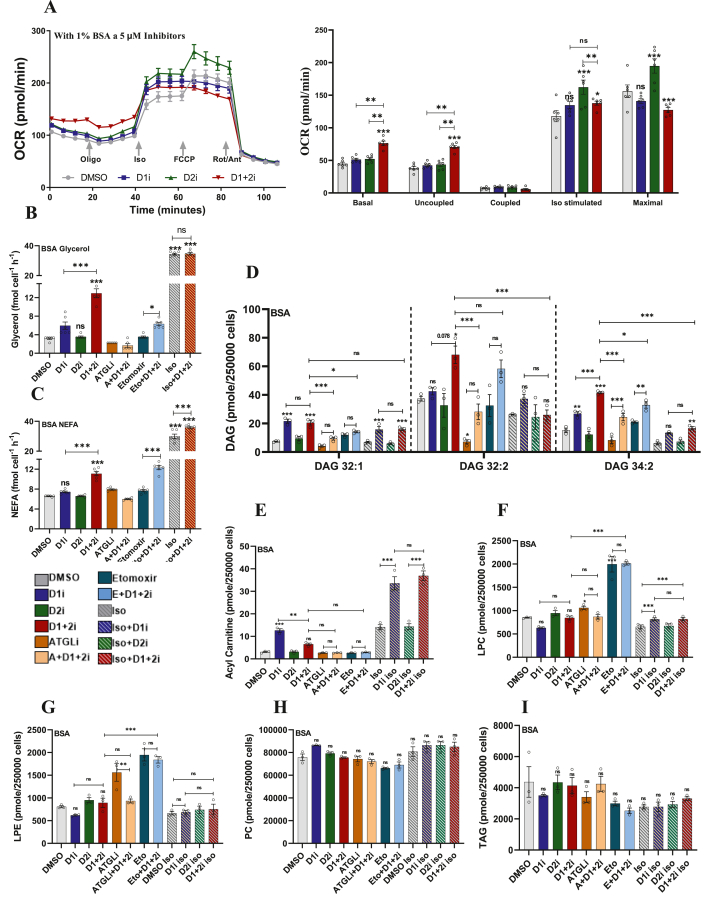
**Effect of DGAT inhibition in the presence extracellular FA quencher (BSA)**. A. OCR measurement and its quantification in the iBA cells pre-treated for 1 h with 2 μ M DGAT inhibitor alone or in combinations in the presence of 1% BSA. Data are mean ± SEM of *n* = 6. Bar graph on the right shows the quantification of the OCR data. B–C. Lipolytic output analysis of (B) free glycerol, and (C) NEFA in iBA adipocytes upon incubation with DGATi in the presence of 1% BSA (*n* = 5–6). D-I. Lipidomic analysis of differentiated iBA adipocytes treated with different pharmacological combination for 2 h in the presence of 1% BSA demonstrate (D) increased basal DAG lipids after D1+2i when compared to D1i. In the iso-stimulated state, DGAT1 is the main DGAT as the D1i is as effective as D1+2i. A similar trend is seen for (E) acyl carnitines suggesting diversion of FA to mitochondria. Other lipid species, (F) LPC, (G) LPE, (H) PC, and (I) TAG showed less prominent alteration. Lipidomic data are presented as mean ± SEM (*n* = 3). A one-way ANOVA with Tukey's post hoc test was applied to test the significance of differences. ns: non-significant; ∗*P* < 0.05; ∗∗*P* < 0.01; ∗∗∗*P* < 0.001.

Our results suggested a large increase in FA utilization upon D1+2i, however, the overall change in total lipid content was minimal (Fig. SI6A). To test if only selected lipid classes are affected by DGATi and to gain insight into the mobilization of key lipid classes, we performed untargeted lipidomic analysis. From all 24 sample groups (*x*3 replicates), we confidently detected/assigned 500+ species of lipids spanning 12 major lipid classes (Figure 5, Figure 6). For ease of presentation, we only show the average of the main lipid classes; the complete dataset can be accessed from the accompanying data file (Dataset1).

Inhibition of DGAT1 (D1i) caused a significant increase in DAG levels (DAG 32:1, DAG 32:2, and DAG 34:2) while D2i did not affect the DAG levels under the basal conditions. The effect of D1+2i was more pronounced than D1i. In contrast, in the iso-stimulated cells, the D1+2i and D1i caused a comparable change in DAG levels that can be attributed to DGAT1 under stimulated conditions but not at the basal level (Fig. 5A). ATGLi treatment led to reduced DAG levels while a combined DGAT and ATGL inhibition (A + D1+2i) led to lower DAGs compared to D1+2i. Although etomoxir alone did not affect DAG levels, Eto+D1+2i led to significantly increased DAG levels, possibly due to reduced mitochondrial usage of activated FAs and its diversion for re-esterification into MAGs to form DAG. Acyl carnitine levels showed a similar pattern of changes wherein basal D1+2i led to higher accumulation than D1i while at iso-stimulated condition D1i was as effective as D1+2i (Fig. 5B). ATGLi reduced the D1+2i-induced acylcarnitine pool while etomoxir fully abolished the D1+2i-induced increase in acylcarnitine levels (Fig. 5B). The basal LPC levels were unchanged by DGAT inhibition, however, under iso-stimulated conditions, D1i and D1+2i increased total LPC levels, possibly as a secondary adaptive mechanism, as etomoxir also led to increased LPC levels (Fig. 5C). Total LPE levels showed a trend similar to LPC (Fig. 5D) although with more pronounced effect under D1i and iso-stimulation. Total PE levels (Fig. SI6B), total PC levels (Fig. 5E), or total TAG levels (Fig. 5F) only showed minimal changes.

Since the study conditions were devoid of any extracellular FA quencher, we next tested if the findings are also reproducible in the presence of BSA (Fig. 6). We first examined the effect of DGATi on the OCR in the presence of 1% BSA. D1i or D2i mildly increased the basal OCR (Fig. 6A), while D1+2i caused a larger increase in OCR, similar to BSA-free conditions. The lipolysis assay further confirmed that D1+2i was more potent than either of the inhibitors alone (Figure 6B,C). Iso treatment induced a strong lipolytic response (Figure 6B,C). Total intracellular FA and glycerol accumulation was comparable across groups (Fig. SI6C,D). The AdipoRed lipid quantification suggested that under basal conditions different treatments did not cause significant changes in the total lipid content while iso treatment slightly depleted the intracellular lipid store (Fig. SI6E) as supported by the increased FA and glycerol accumulation in the media. We next performed a lipidomic analysis which led to the conclusion that except for a few differences, overall changes in cellular lipid species were similar to that seen in the BSA-free conditions. For instance, in the presence of BSA, the accumulation of DAG 32:1 (but not of DAG 32:2 or DAG 34:2) was comparable between D1i and D1+2i (Fig. 6D). Also, the levels of acylcarnitines in D1+2i treated cells were significantly lower than D1i, possibly due to increased intracellular FA turnover to compensate for the OCR and the FA quenching by BSA (Fig. 6E). Other lipid species, LPC (Fig. 6F) and LPE (Fig. 6G) followed trends similar to the ones described for BSA-free conditions, while PC (Fig. 6H), TAG (Fig. 6I), and PE (Fig. SI6F) were largely unperturbed. Overall, the lipidomic analysis suggests that inhibition of re-esterification causes a selective change in cellular lipid profile to accommodate excess FA while maintaining the cellular lipidomic balance.

### 3.6 AMPK activation and mitochondrial pyruvate influx mediate increased OCR

**Figure 7:**
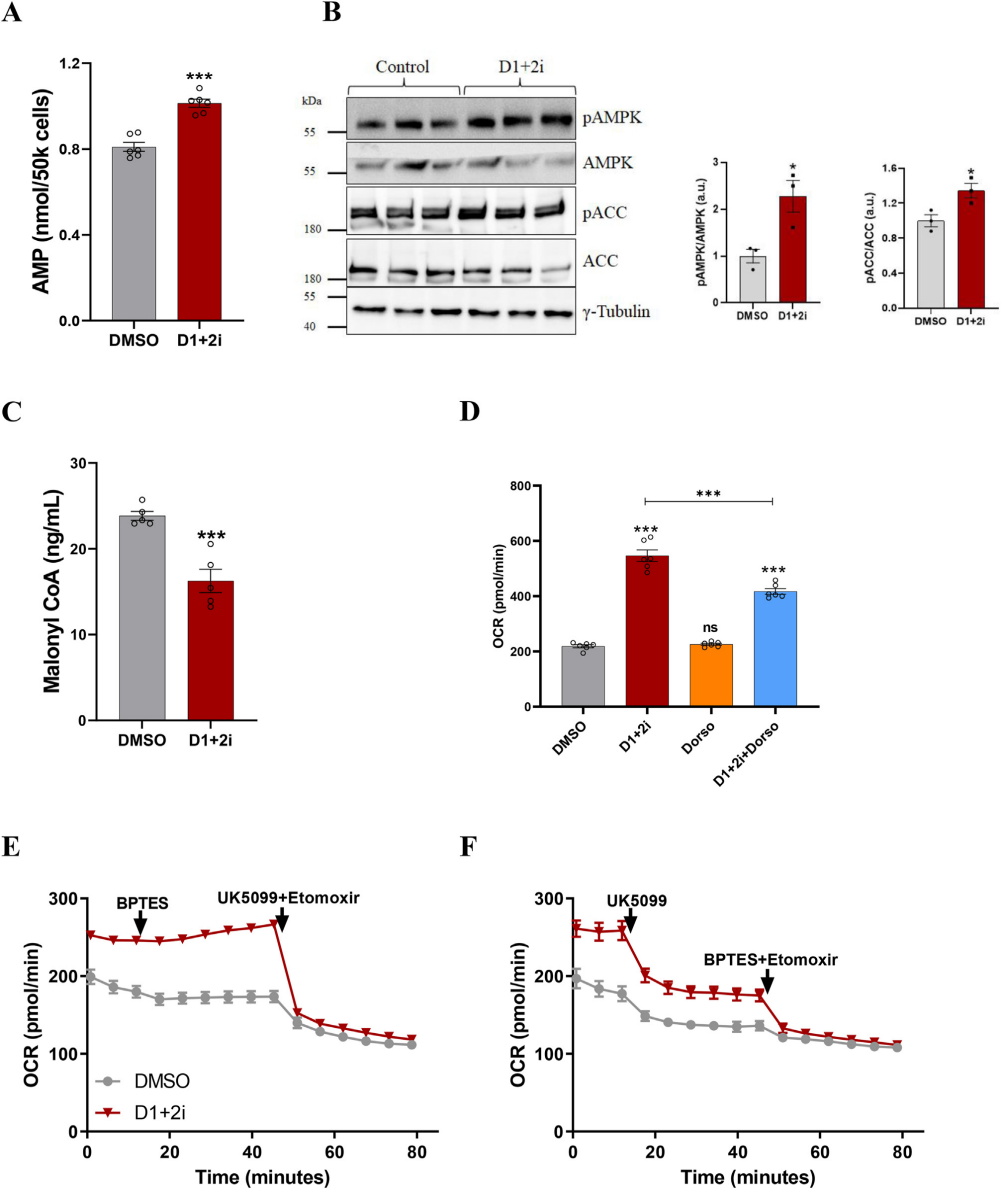
**Concurrent AMPK activation and mitochondrial pyruvate influx mediate increased OCR**. A. AMP levels in iBA adipocytes treated with vehicle or D1+2i (*n* = 6). B. Western blots showing the change in AMPK and ACC phosphorylation D1+2i treatment (n = 3). Blots on right show a representative chemiluminsence image and bar graphs on the right show densitometric quantification done using Image lab 6.0 software. C. Malonyl CoA levels in iBA adipocytes treated with vehicle or D1+2i (*n* = 4). D. OCR from the samples treated with DMSO or D1+2i in the presence/absence of AMPK inhibitor (Dorsomorphin) (*n* = 6). E-F. Substrate dependence of D1+2i induced OCR in iBA cells. The iBA adipocytes were pretreated for 1 h with DMSO or D1+2i (*n* = 5). The metabolic inhibitors were auto-injected in the given order. All the data are presented as mean ± SEM. For two group comparisons in A, B, and C, a two-tailed unpaired t-test was applied to test the significance of differences. For the data presented in D, one-way ANOVA with Tukey's post hoc test was applied to test the significance of differences. ∗*P* < 0.05; ∗∗∗*P* < 0.001.

To delineate the mechanism behind the shuttling of palmitoylcarnitine upon D1+2i at basal levels, we considered alternate possibilities. We examined AMP levels that would arise as a by-product of uncoupling and observed that at the basal level, D1+2i led to a significant increase in AMP levels (Figure 7A). AMP is the prime activator of AMPK, and we could also show an increased AMPK phosphorylation upon D1+2i (Figure 7B; Fig. SI6G). ACC is an important downstream target of AMPK that regulates de novo lipogenesis and AMPK-mediated inhibitory phosphorylation has been shown to lead to a reduction in malonyl CoA [32], which in turn would modulate mitochondrial FA import. We thus measured the ACC phosphorylation which was significantly increased while malonyl CoA levels were significantly decreased (Figure 7B,C), which corroborates with the increased mitochondrial utilization of FAs. To further validate the involvement of the AMPK activation in mediating the OCR surge, we performed OCR measurement in the presence of compound C (Dorsomorphin, AMPKi) a specific inhibitor of AMPK. We found that AMPKi blunted the D1+2i-induced OCR by ∼40% (Figure 7D; Fig. SI6H). The effect of AMPKi was D1+2i specific as we did not observe any change in OCR levels by AMPKi in control cells. Since AMPK was responsible only for ∼40% OCR, we analyzed the dependence of the OCR increase on glutamine and pyruvate using BPTES (glutaminase inhibitor) or UK5099 (mitochondrial pyruvate carrier inhibitor). While BPTES did not affect the observed changes in OCR, UK5099 diminished the D1+2i-induced OCR by ∼40% (Figure 7E,F). Together, these results suggest that AMPK-mediated mitochondrial FA import and independent pyruvate utilization might contribute partly to the energetic demands of increased OCR upon inhibition of re-esterification.

## 4 Discussion

Lipid metabolism lies at the heart of whole-body energy homeostasis. Owing to high energy equivalence, TAGs are the preferred storage molecule [33], however, excessive lipid deposition in adipose tissue, ectopic lipid accumulation in non-adipose organs, or an increase in circulating FA/TAG levels are important contributors to systemic insulin resistance [[34], [35], [36], [37], [38]]. Therefore, besides the absorption of ingested meal-derived lipids and their storage as TAGs, perpetual basal lipolysis during the post-absorptive phase and subsequent re-esterification of spare FA seems to be relevant to ensure a fine-tuned lipid balance.

*Dgat1* shows a broad tissue distribution, while *Dgat2* seems to be more specific to organs with substantial lipid turnovers such as the liver and the adipose tissue [11]. Interestingly, *Dgat1* is prominently expressed in the gastrointestinal tract and thus is implicated in meal-derived lipid absorption [15,17,[39], [40], [41]]. Based on the improved metabolic function observed in *Dgat1* knockout mice, it was proposed that DGAT1 inhibitors could be an effective therapeutic strategy to counter metabolic disorders [17]. Chronic treatment with DGAT1 inhibitors recapitulated in part the beneficial effects of *Dgat1* KO and improved insulin sensitivity & glycaemic balance [[42], [43], [44], [45]]. Given the prominence of *Dgat1* (over *Dgat2*) in the GI tract and considering reduced tissue fat in *Dgat1* KO or the inhibitor-treated animals, it is believed that most of the lipid-related effects of *Dgat1* KO originate from the inhibition of fat absorption. Despite the clear distinction of DGAT1 in esterifying the majority of lipolysis-derived FAs, there seems to be some specialization of the function of DGAT1 and 2 possibly in a species and tissue-dependent manner [8,[10], [11], [12],^22^,^33^]. *DGAT1* loss-of-function mutation in humans causes severe diarrhea [46] but a global *Dgat1* knockout or pharmacological inhibition of DGAT1 in mice does not exhibit a similar phenotype [14,18,35], while, a combined DGAT inhibition in mice causes diarrhea-like symptoms [15]. Thus, it seems that intestinal absorption in humans depends on DGAT1, while in mice DGAT2 can in part replace intestinal DGAT1 action.

While studying the key enzyme responsible for the re-esterification of lipolytic FA in 3T3 L1 adipocytes, Chitraju et al., demonstrated the prominence of DGAT1 over DGAT2 during iso-stimulated lipolysis [6]. However, at the basal level-only a combined inhibition (but not the individual DGAT inhibition), significantly impacted re-esterification [6], pointing towards overlapping actions of DGAT1 and DGAT2. Consistent with previous findings, we observed a more pronounced inhibitory response upon DGAT1 inhibition than with DGAT2 [6,8]. Combined inhibition of DGAT1 and 2 led to a large increase in OCR which was mainly driven by mitochondrial FA import. Substrate preference of DGAT1/2 may explain this observation. DGAT2 was shown to preferentially use ATGL-derived DAG as substrate [47] and thus might only partially compensate for DGAT1 activity [6,9,13,40]. Similarly, the localization might explain the observed phenomenon as unlike DGAT1 (which is an ER-resident enzyme), DGAT2 also localizes to mitochondria [48]. One possible explanation is that DGAT1 re-esterifies FAs on the endoplasmic reticulum near the LD release site and since DGAT1 is the main isoform, DGAT1 inhibition would divert fatty acyl CoA to mitochondria. In contrast, DGAT2 could partly re-esterify excess FAs, which could account for the small increase in OCR observed upon DGAT1 inhibition. Besides, as reported in HepG2 cells, increased stability or activity of DGAT2 upon DGAT1 inhibition is also a possibility [49].

Complete hydrolysis of one TAG molecule generates one glycerol molecule and three FAs. In our analyses, the FA levels were disproportionately low and the stoichiometric ratio of glycerol:FA (1:3) was never achieved. Based on extracellular glycerol, DGAT1 inhibition or a combined DGAT1/2 inhibition caused higher lipolysis/glycerol efflux than control or DGAT2 inhibition. Since this study was performed in the absence of a FA quencher, we also measured intracellular FA levels to rule out intracellular uptake/retention. A comparable amount of intracellular FA suggests the terminal utilization of excess FAs upon DGAT1+2 combined inhibition. Another puzzling observation was an increase in glycerol release after ATGLi + DGAT1+2 inhibition compared to ATGLi alone (Fig. 2D). We speculate that this might originate from glucose-derived G-3-P or the residual lipolysis from ATGL/HSL or other mechanisms. Nevertheless, the extent of the increase was much smaller than D1+2i without ATGLi, highlighting the ATGL-mediated lipolytic contribution.

To derive insight into the fate of FAs upon DGAT1/2 inhibition, we performed ^13^C-palmitate tracing. We observed a similar lipidomic profile of key glyceride species (DAG/TAG/Acylcarnitines) as reported previously [8]. Regarding the difference in the magnitude of incorporation of ^13^C-palmitate to DAG at basal vs iso-stimulated conditions, we speculate that complete hydrolysis of TAGs at basal conditions generates MAGs and FA which are readily converted to DAGs. In contrast, iso-stimulation causes only partial lipolysis of TAGs to DAGs, and thus the ^13^C-palmitate labeling of DAGs is reduced. Upon DGAT1 inhibition, the DAGs that would have been esterified to TAG by DGAT1 are possibly further hydrolyzed into MAGs, which could account for the observed enhanced labeling. Besides, untargeted lipidomic analysis of endogenous lipids showed that DGATi leads to the accumulation of endogenous DAG and acylcarnitine species. So, a likely explanation is that the ^13^C containing DAG and acylcarnitines levels increase. However, due to a proportional increase in labeled and unlabelled DAG and acylcarnitines, the fractional labeling remains constant.

A previous study reported transcriptional changes upon D1+2i [6]. However, we did not observe any substantial transcriptional alterations in the majority of the tested genes at basal conditions. One explanation could be the shorter inhibitor incubation time used here. Under iso-stimulation, a trend towards a decreased expression of lipid storage genes was observed when both DGATs were inhibited, possibly due to increased energy demand. Although a clear role of AMPK is evident, we speculate that D1+2i treated adipocytes possibly adapt to release the CPT1 inhibition through a reduction in malonyl CoA levels via AMPK activation.

Mitochondrial pyruvate influx appears to play a crucial role in the energy production of unstimulated brown adipocytes. Mitochondrial pyruvate carrier (MPC) inhibition is shown to increase the lipolysis coupled re-esterification cycling [50]. Pyruvate is a key substrate for the tricarboxylic acid cycle (TCA cycle) as a source of oxaloacetate or acetyl-CoA. It appears that after D1+2i treatment, the increased influx of mitochondrial FAs serves as a powerful uncoupler. The pyruvate-fueled TCA cycle, in conjunction with FAO, likely plays a role in maintaining a healthy proton gradient. Moreover, given the dynamic exchange of acetyl-CoA, the role of pyruvate in de novo FA synthesis is also a possibility.

A recent pre-print study analyzed the effect of adipose-specific Dgat1/Dgat2 double knockout (aDKO). The aDKO mice on HFD show substantially higher energy expenditure and reduced RER corroborating with our data of increased OCR and FAO. These data provide physiological credence to our findings and suggest that re-esterification may be a fundamental mechanism that also fine-tunes fuel utilization and FAO [24].

Besides these interesting observations, this study has several limitations that should be considered when interpreting the results. It is an *in vitro* study performed under defined conditions. Therefore, although major findings seem to translate to physiological context as discussed above [24], some results may be condition-specific and thus should be interpreted accordingly. In addition, since some other non-adipocyte cell types have considerable lipid stores, it is worth exploring if the inhibition of re-esterification has a similar effect in other cells [51]. In the physiological context, discerning tissue-specific and systemic effects of blockage of re-esterification (using a pharmacological approach and tissue-specific conditional knockouts) therefore is an important next step. Another interesting finding from the study is the accumulation of various DAG species following DGAT inhibition. As different DAG species serve as precursors for many (phospho)lipid species, and as direct signalling molecules and its availability and location can affect the activity of protein kinase C/D (PKC/PKD), it would be worthwhile to investigate the impact of DAG-mediated PKC signalling in cells/tissue after DGAT inhibition.

Our findings demonstrate a continuous cycle of re-esterification of FA to glyceride species at basal as well as stimulated lipolytic conditions. Given a high ATP equivalent cost of FA re-esterification [52], an energetic consequence could be relevant for whole-body energy homeostasis.

## Funding

The work was supported by the 10.13039/100000001Swiss National Science Foundation (SNSF; to C·W.) and FreeNovation grant (Novartis; to C·W.).

## Author contribution

A.K.S. and C.W. conceived the study. A.K.S. designed and performed all the experiments and analysis with the help from T.W., R.K., S.M. and M.B. T.W. contributed considerably in primary cell isolation. A.O. and N.Z. performed mass-spec analysis. C.W. supervised and acquired all the funding. A.K.S. and C.W. wrote the manuscript. All authors approved the manuscript.

## Data Availability

Dataset is provided
