# Supplementary material for: Basal re-esterification finetunes mitochondrial fatty acid utilization
Source: Mol Metab. 2023 Mar 4;71:101701. doi: 10.1016/j.molmet.2023.101701 (PMC10011057; doi:10.1016/j.molmet.2023.101701)
Supplement: Multimedia component 1 [file mmc1.pdf]

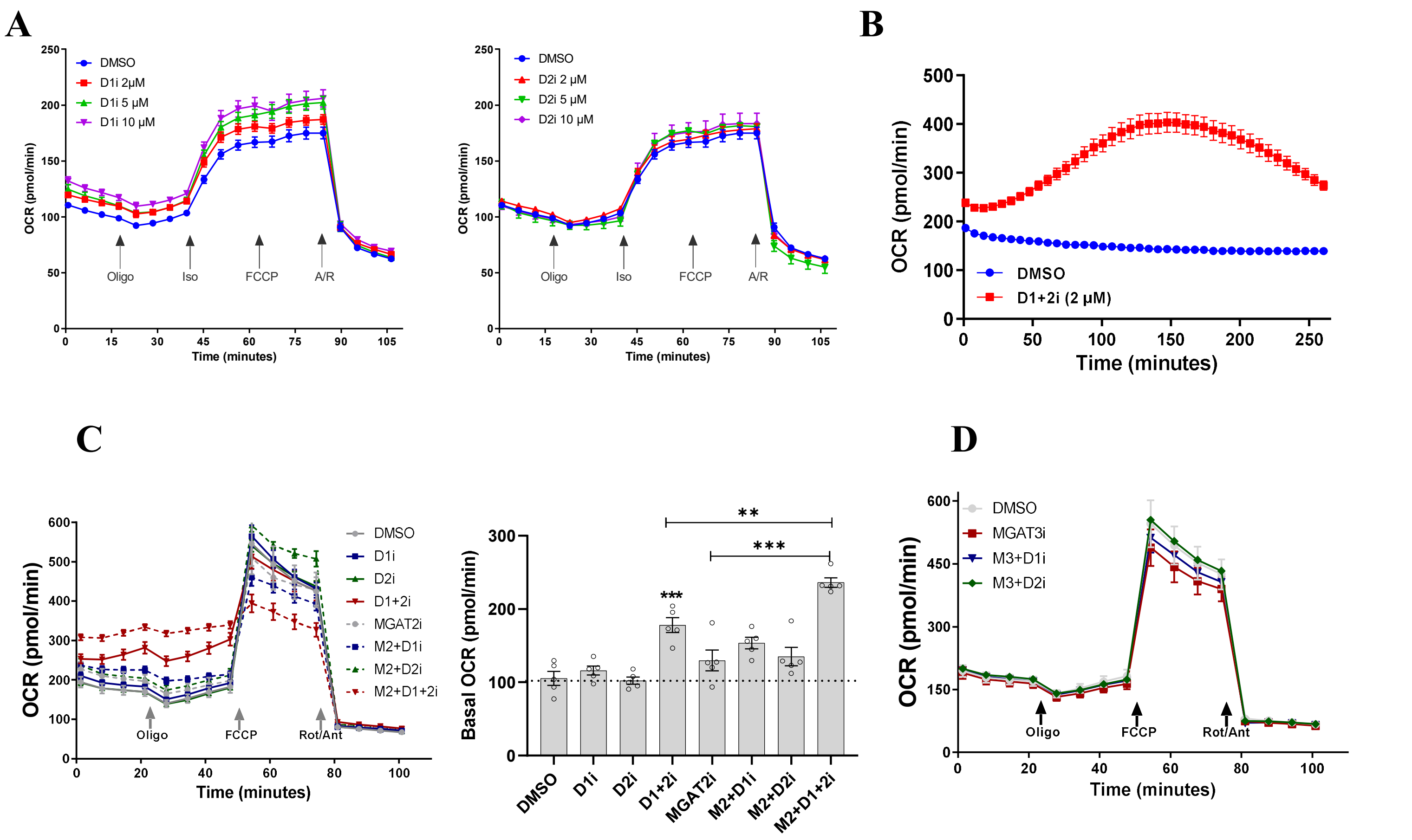

Figure SI1: MGAT2i enhances DGATi induced OCR

A. Dose optimization of DGAT inhibitors. These Inhibitors were frequently used before in several high-quality studies (e.g., Chitraju et al, Cell Metabolism, 2017; Nguyen et al., Developmental Cell, 2017). Chitraju et al used 5 µM inhibitor concentrations while Nguyen et al used 20 µM DGAT1i (T863) and 10 µM DGAT2i. We took these concentrations as reference points and tested the dose response of individual inhibitors. We observed that 2 µM inhibitor concentrations were enough when the experiments were performed in the BSA free condition. In the absence of BSA, 2 µM DGAT1i induced an increase in OCR while 5 µM dose was comparable to 2 µM dose. A substantially increased 10 µM dose still slightly increased the OCR, however, considering the possible toxicity at high doses, we proceeded with the 2 µM dose. For the experiments in the presence of BSA 5 µM inhibitor concentrations was optimal.

B. Optimization of incubation time for DGAT inhibitor for differentiated iBA adipocytes. Cells were incubated with inhibitors and kept in CO2 free incubator for 40 minutes followed by the OCR measurement for >4 hours. OCR response peaked at ~2.5 hours and remained higher than the control group until the end of the measurement. Thus, the time point chosen for the terminal experiments (2 hours post incubation) seems ideal as it avoids saturation, while still being in the incremental phase.

C. OCR measurement in the iBA cells pre-treated for 1 hour with 5 µM DGAT1/2 and/or MGAT2 inhibitors (Cpd24d, calbiochem) alone or in combinations. Data are mean ± SEM (*n* = 6). Bar graph on right shows the basal OCR of respective groups.

D. OCR measurement in the iBA cells pre-treated for 1 hour with 5 µM DGAT1/2 and/or MGAT3 inhibitor (PF-06471553) alone or in combinations. Data are mean ± SEM (*n* = 6).

A one-way ANOVA with Tukey's post hoc test was applied to test the significance of differences. $*P < 0.05$.

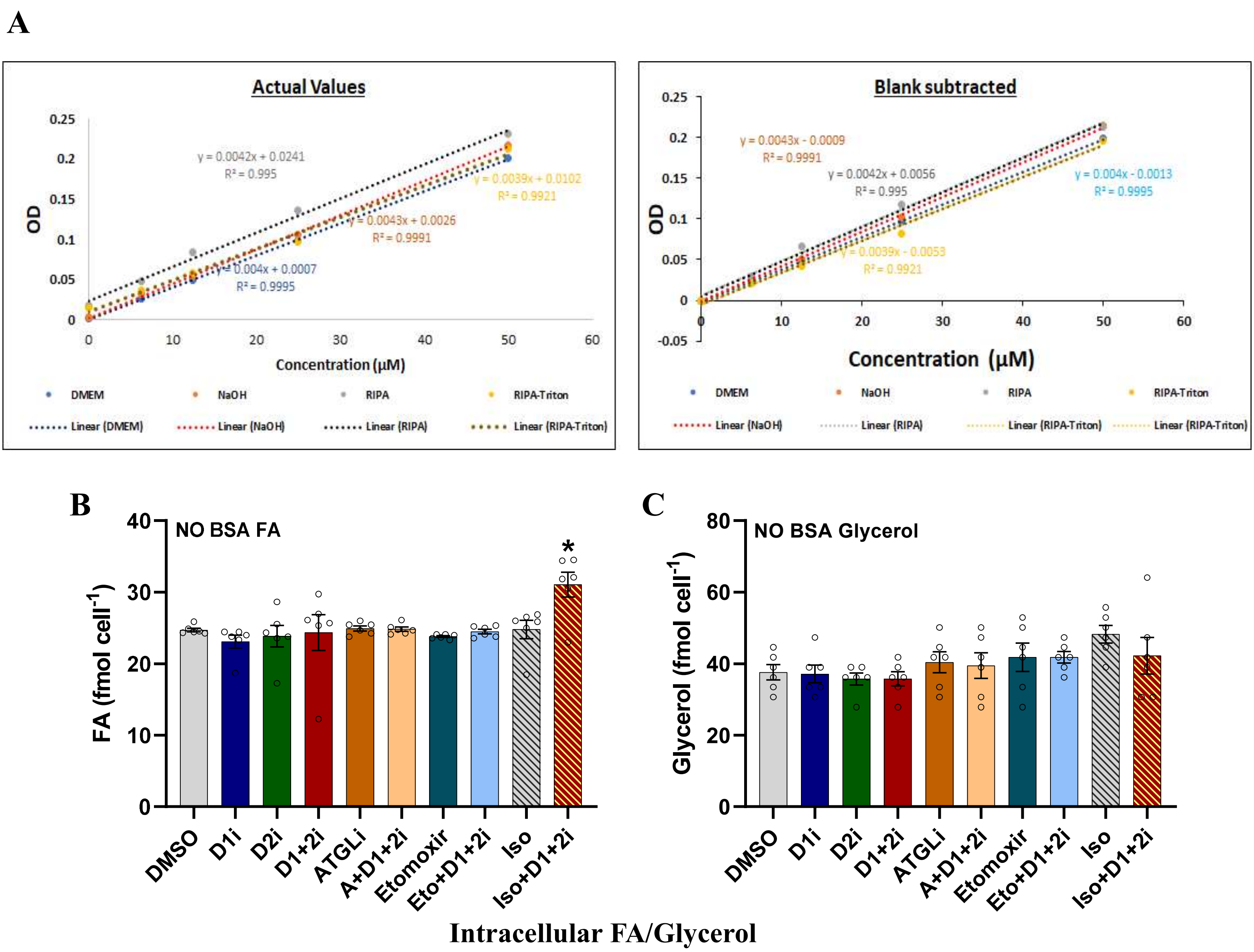

Figure SI2. Intracellular FA /glycerol measurement

A. Testing lysis buffers that minimally interfere with intracellular NEFA measurement. We tested the effect of different lysis solutions on the performance of the kit. We used serum/phenol red-free DMEM as a reference buffer (used in lipolysis assays for extracellular NEFA) and tested the effect of lysis buffers (i) 50 mM NaOH (ii) standard RIPA buffer (iii) or triton X-100 free RIPA. We used the NEFA standard in these solutions to plot OD vs concentration plot to assess the compatibility. We noticed that the use of RIPA led to a marginal background reading (positive value even in the blank) and the deviation was <7%. When we performed a blank subtraction, the deviation was ~2%. Using a triton-free RIPA did not improve the background while it slightly compromised the lysis; therefore, we used standard RIPA for the lysis.

B. Intracellular FA levels in iBA cells upon incubation with DGATi showing comparable accumulation of NEFA after different inhibitor treatments (n = 6).

C. Intracellular glycerol levels in iBA cells upon incubation with DGATi showing comparable accumulation of NEFA after different inhibitor treatments (n = 6).

For the data shown in Fig SI2B, C, a one-way ANOVA with Tukey's post hoc test was applied to test the significance of differences. *$P < 0.05$.

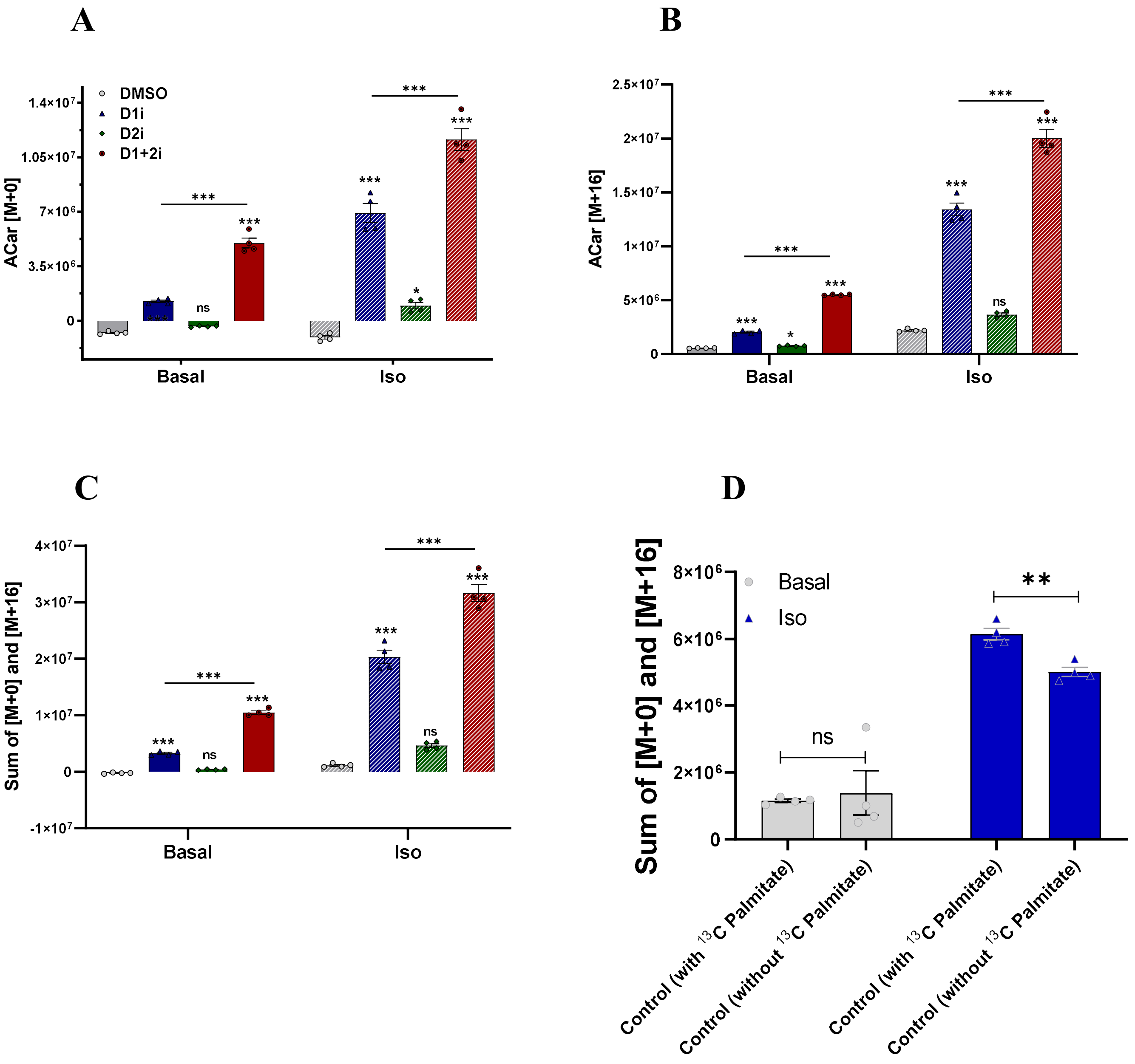

Figure SI3: D1+2i increases acylcarnitine pool

A-C. Quantification of intensity of (A) unlabled acylcarnitine [M+0], (B) ^{13}C labelled acylcarnitine [M+16], and (C) sum of labelled and unlabelled isotopologues of acylcarnitine [(M+0) + (M+16)]. The data are represented as blank (no ^{13}C-palmitate) subtracted values ± SEM (n=4). one-way ANOVA with Tukey's post hoc test was applied to test the significance of differences. ns: non-significant; *$P < 0.05$; ***$P < 0.001$.

D. Sum of (M+0) and (M+16) isotopologues in controls groups incubated with/without ^{13}C palmitate (n=4). The data represent mean ± SEM (n=4). A two-tailed unpaired t-test was applied to test the significance of differences. ns: non-significant; **$P < 0.01$.

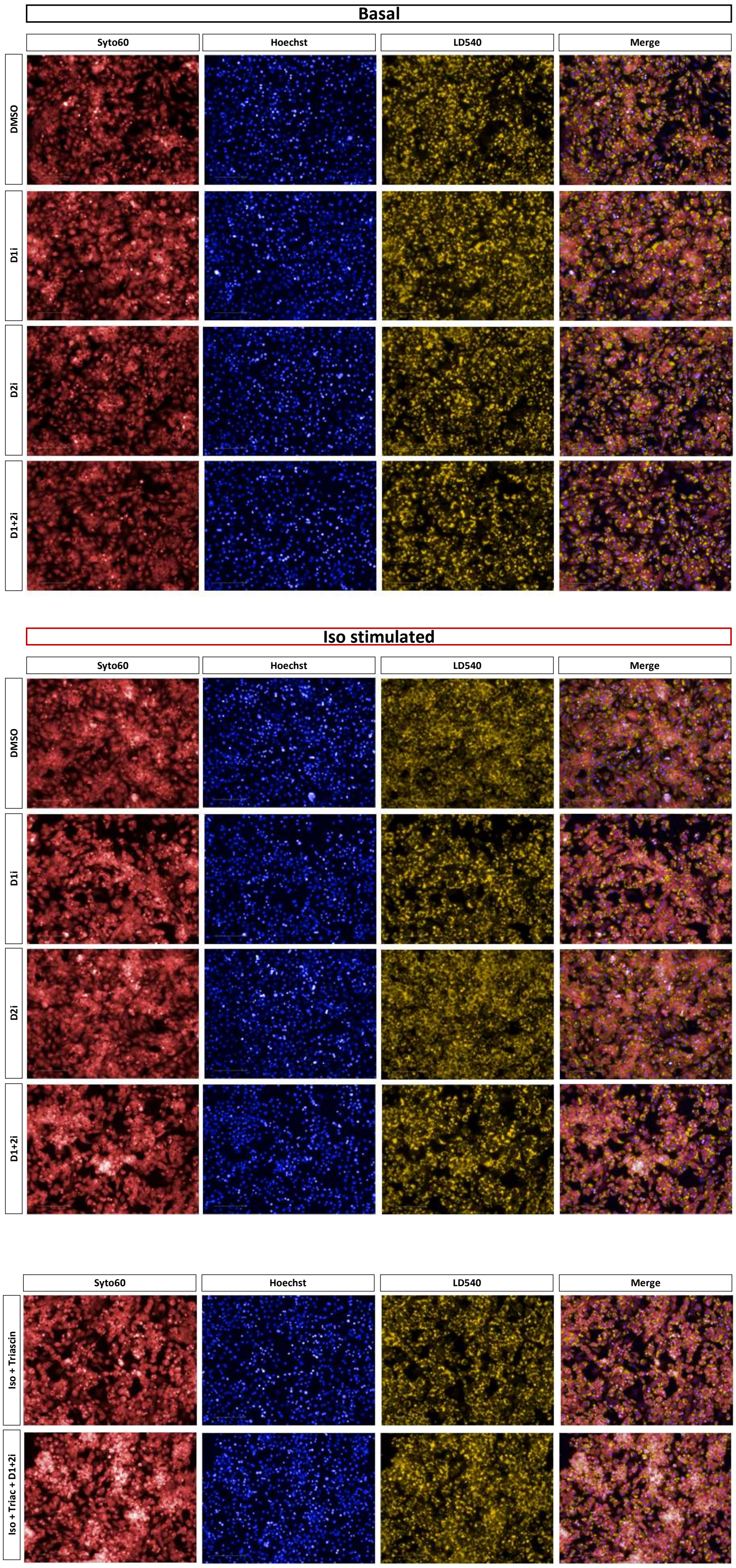

Figure SI4: DGATi induced changes in lipid droplet abundance/size

A. Representative microscopic images of iBA cells used to calculate LD parameters shown in Figure 4B, C.

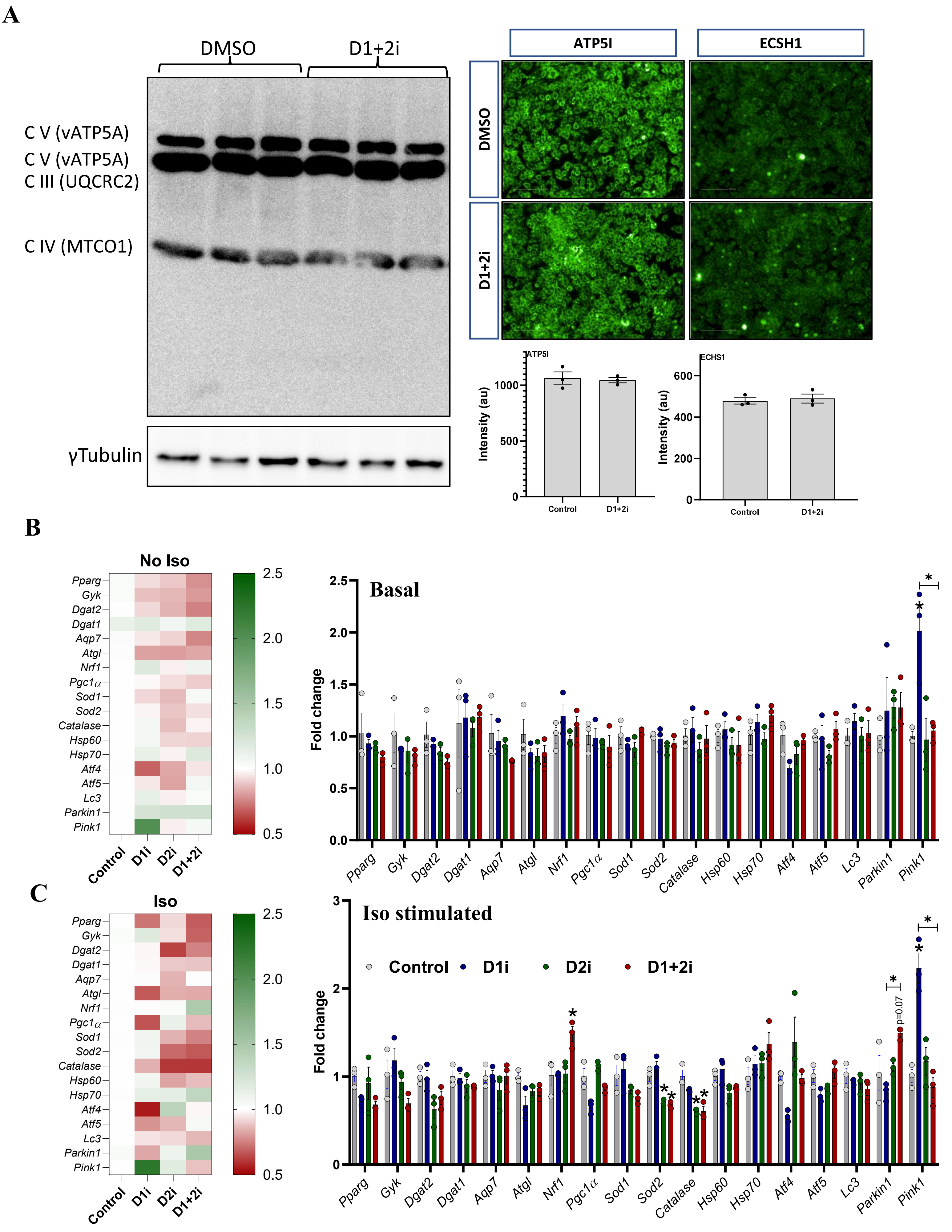
A
DMSO
D1+2i
C V (vATP5A)
C V (vATP5A)
C III (UQCRC2)
C IV (MTCO1)
γTubulin
ATP5I
ECSH1
DMSO
D1+2i
ATP5I
ECHS1
Intensity (au)
0
500
1000
0
200
400
600
Control
D1+2i
B
No Iso
Pparg
Gyk
Dgat2
Dgat1
Aqp7
Atgl
Nrf1
Pgc1α
Sod1
Sod2
Catalase
Hsp60
Hsp70
Atf4
Atf5
Lc3
Parkin1
Pink1
Control
D1i
D2i
D1+2i
2.5
2.0
1.5
1.0
0.5
Basal
Fold change
0.0
0.5
1.0
1.5
2.0
2.5
C
Iso
Iso stimulated
Control
D1i
D2i
D1+2i
p=0.07
0
1
2
3

Continued….

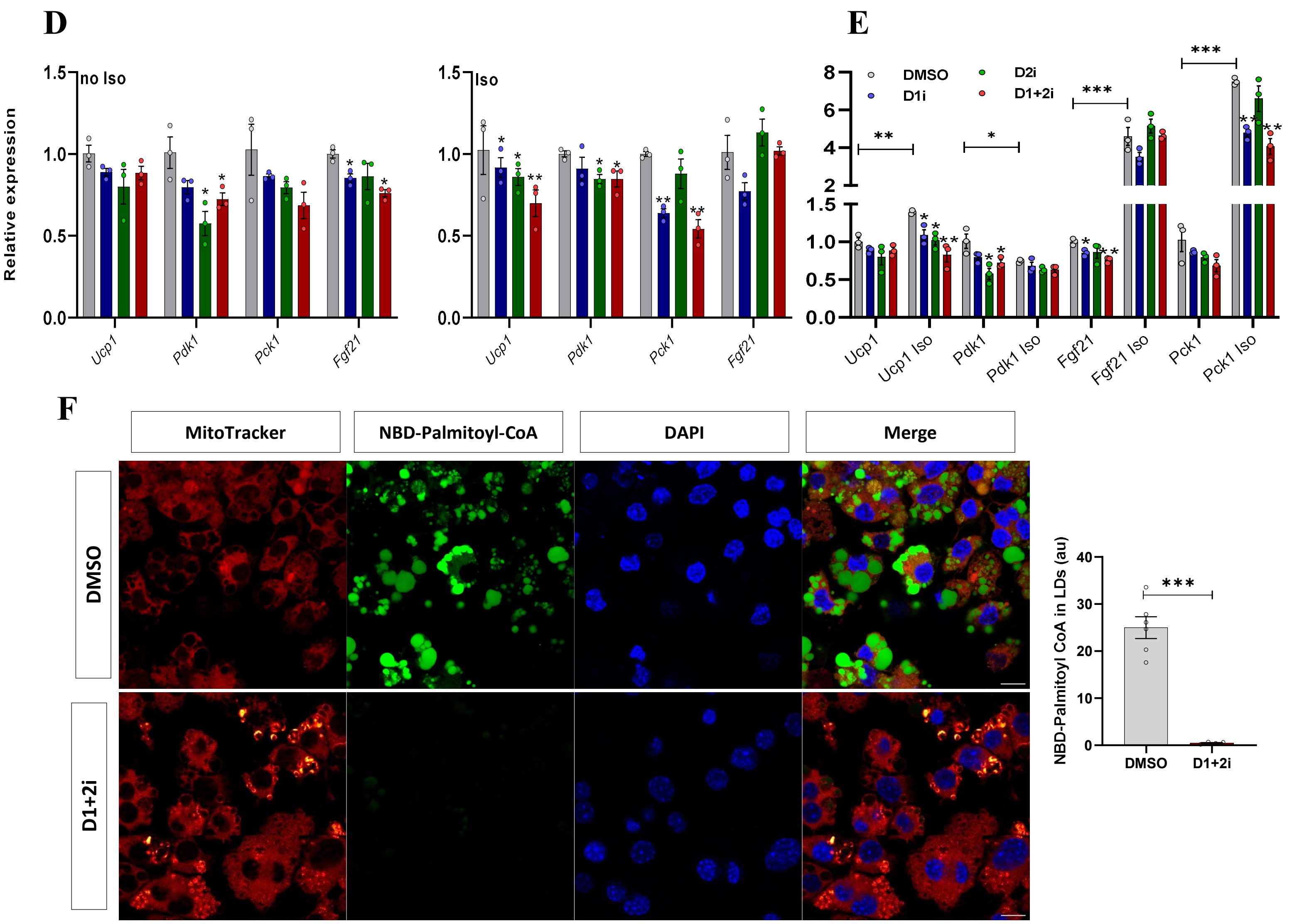

Figure SI5: Acute DGATi block esterification of NBD-palmitoyl-CoA and minimally affect the genes expression in iBAs

A. Quantification of mitochondrial protein expression. D1+2i does not affect mitochondrial protein abundance. WB on the left shows that key OXPHOS protein levels remain unperturbed by D1+2i. On the right side, representative mages (top) and fluorescence intensity quantification (bottom) showing that mitochondrial ATP5I and ECHS1 protein levels remain unperturbed by D1+2i.

B. Relative mRNA expression of selected genes in DGATi treated iBAs at basal state. Left: heatmap, right: bar graph of the relative gene expression (n = 3).

C. Relative mRNA expression of selected genes in DGATi treated iBAs in isoproterenol stimulated state. Left: heatmap, right: bar graph of the relative gene expression (n = 3). Heat maps are plotted for the mean of the values. For the bar graph, data are presented as mean ± SEM (n = 3). For the datasets in Fig. SI4A, and B, one-way ANOVA with Tukey's post hoc test was applied to test the significance of differences. *$P < 0.05$.

D. Modulation of the expression of Ucp1, Pdk1, Pck1, and Fgf21. Data are expression is relative to the respective control controls (left graph: no iso; right graph: in the presence of iso).

E. Modulation of the expression of Ucp1, Pdk1, Pck1, and Fgf21 when the expression data are normalized relative to the basal control group.

F. Microscopic images showing the effect of D1+2i on the extent of incorporation of NBD-palmitoyl CoA into LDs. The panel on the right shows total cellular NBD fluorescence in control vs D1+2i. Differentiated iBA adipocytes were first treated with DMSO/D1+2i for 45 min followed by addition of 100 μM NBD-palmitoyl CoA and mitotracker red. After 15 min, cells were washed 3x and fixed for microscopic observation. Data are presented as mean ± SEM (n = 4-5). A two-tailed unpaired t-test was applied to test the significance of differences. ns: non-significant; ***$P < 0.001$.

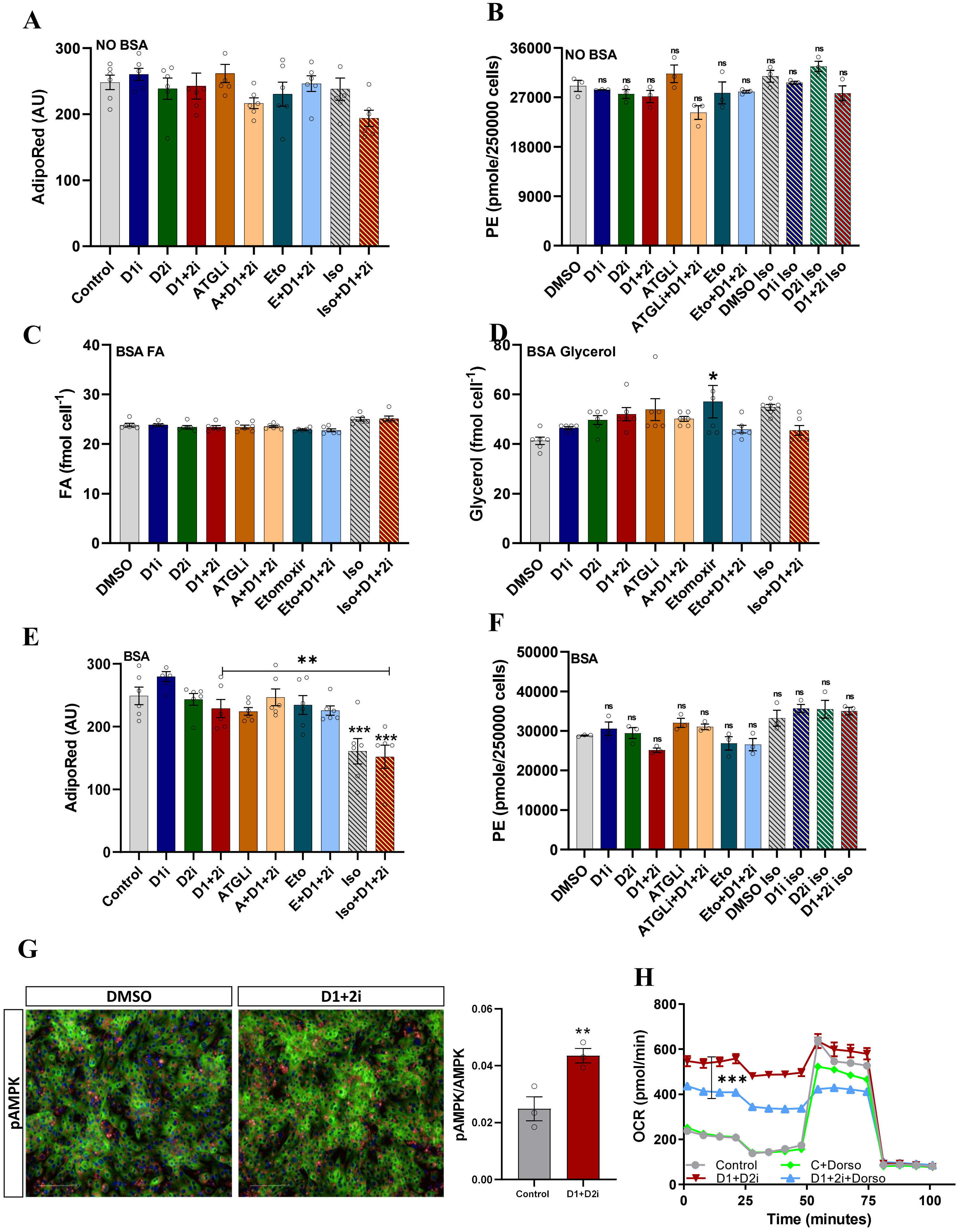
A
NO BSA
AdipoRed (AU)
Control
D1i
D2i
D1+2i
ATGLi
A+D1+2i
Eto
E+D1+2i
Iso
Iso+D1+2i
B
NO BSA
PE (pmole/250000 cells)
DMSO
D1i
D2i
D1+2i
ATGLi
ATGLi+D1+2i
Eto
Eto+D1+2i
DMSO Iso
D1i Iso
D2i Iso
D1+2i Iso
C
BSA FA
FA (fmol cell-1)
Etomoxir
D
BSA Glycerol
Glycerol (fmol cell-1)
E
BSA
F
BSA
G
DMSO
D1+2i
pAMPK
pAMPK/AMPK
Control
D1+D2i
H
OCR (pmol/min)
Time (minutes)
Control
D1+D2i
C+Dorso
D1+2i+Dorso

Figure SI6: D1+2i stimulates AMPK that partly mediated OCR

A. Total cellular lipid content as measured by AdipoRed after 2 hours treatment with respective combination in the absence of BSA showing minal changes by these treatments (n = 6).

B. PE levels in differentiated iBA adipocytes treated with different pharmacological combination for 2 hours in the absence showing minimal changes on the overall PE levels (n = 3).

C. Intracellular fatty acid levels upon incubation respective treatments showing comparable accumulation of fatt acids after different inhibitor treatments in the presence of BSA (n = 6).

D. Intracellular glycerol levels upon incubation respective treatments showing comparable accumulation of glycerol after different inhibitor treatments in the presence of BSA (n = 6).

E. Total cellular lipid content as measured by AdipoRed after 2 hours treatment with respective combination in the presence of BSA (n = 6).

F. PE levels in differentiated iBA adipocytes treated with different pharmacological combination for 2 hours in the absence showing minimal changes on the overall PE levels (n = 3).

G. Representative microscopic images of pAMPKα immunostaining from operetta showing increased levels of pAMPK after D1+2i treatment. The mean fluorescence intensity is presented in the bar graph on the right (n = 3).

H. Seahorse OCR measurement graph showing the effect of Dorso (AMPK inhibitor) on D1+2i induced OCR. The quantitative data is presented in the figure 7C. The data are presented as mean ± SEM (n = 6).

All data are presented as mean ± SEM. Except for the data shown in EV5G (where a two tailed unpaired t-test was applied), one-way ANOVA with Tukey's post hoc test was applied to test the significance of differences. *$P < 0.05$; **$P < 0.01$; ***$P < 0.001$.
